# Machine learning approaches for biomarker discovery to predict large-artery atherosclerosis

**DOI:** 10.1038/s41598-023-42338-0

**Published:** 2023-09-13

**Authors:** Ting-Hsuan Sun, Chia-Chun Wang, Ya-Lun Wu, Kai-Cheng Hsu, Tsong-Hai Lee

**Affiliations:** 1 Artificial Intelligence Center, China Medical University Hospital, Taichung, Taiwan; 2 Department of Neurology, China Medical University Hospital, Taichung, Taiwan; 3 Department of Medicine, China Medical University, Taichung, Taiwan; 4 Stroke Center and Department of Neurology, Linkou Chang Gung Memorial Hospital, and College of Medicine, Chang Gung University, Taoyuan, Taiwan

**Keywords:** Cardiovascular diseases, Biomarkers, Risk factors, Mathematics and computing, Cardiovascular biology

## Abstract

Large-artery atherosclerosis (LAA) is a leading cause of cerebrovascular disease. However, LAA diagnosis is costly and needs professional identification. Many metabolites have been identified as biomarkers of specific traits. However, there are inconsistent findings regarding suitable biomarkers for the prediction of LAA. In this study, we propose a new method integrates multiple machine learning algorithms and feature selection method to handle multidimensional data. Among the six machine learning models, logistic regression (LR) model exhibited the best prediction performance. The value of area under the receiver operating characteristic curve (AUC) was 0.92 when 62 features were incorporated in the external validation set for the LR model. In this model, LAA could be well predicted by clinical risk factors including body mass index, smoking, and medications for controlling diabetes, hypertension, and hyperlipidemia as well as metabolites involved in aminoacyl-tRNA biosynthesis and lipid metabolism. In addition, we found that 27 features were present among the five adopted models that could provide good results. If these 27 features were used in the LR model, an AUC value of 0.93 could be achieved. Our study has demonstrated the effectiveness of combining machine learning algorithms with recursive feature elimination and cross-validation methods for biomarker identification. Moreover, we have shown that using shared features can yield more reliable correlations than either model, which can be valuable for future identification of LAA.

## Introduction

Large-artery atherosclerosis (LAA) is a pathological condition characterized by the formation of chronic plaques in arteries, which can lead to obstructed blood flow and resulting in ischemic injury. LAA is a multifactorial disease responsible for 20–30% of ischemic stroke cases^1^. Clinically, non-invasive examinations such as ultrasound, computed tomography (CT), and magnetic resonance angiography (MRA) are typically used to confirm the diagnosis. However, these tests are often expensive and time-consuming, and their accuracy may be dependent on the skill level of the technician performing the exam. Therefore, there is an urgent clinical need to identify novel and more efficient biomarkers for predicting the risk of LAA, which can be achieved through the general blood tests.

Well-known risk factors include age, gender, and family history of stroke, hypertension, diabetes, hyperlipidemia, obesity, alcohol consumption, and tobacco smoking^2^. Studies have also found that endothelial dysfunction and the resulting inflammatory response may lead to compromised endothelial integrity and plaque formation^3–8^. Altered metabolism is a hallmark of acute myocardial ischemia, providing more real-time cell signaling information than other clinical symptoms^9–11^. Several pathways, particularly cholesterol, purine, pyrimidine, and ceramide pathways^12–14^, are found to be altered when atherosclerosis occurs. These molecules were considered being the novel biomarkers and therapeutic targets for LAA.

In addition to the complexity and heterogeneity of LAA pathology, the dynamic nature of metabolism also makes traditional statistical methods ineffective for such large and complex data sets. Machine learning (ML) approaches have shown promising in diagnosis improvement, risk prediction, and disease treatment for chronic cardiovascular diseases based on lifestyle^15^, biochemical testing^16^, electrocardiograms^17^, medical imaging^18^, and genetic, genomic, and proteomic biomarkers^19^. For example, a study in India used ML algorithms to automatically identify and quantify carotid artery plaques in MRI scans. They achieved 91.41% accuracy in LAA classification using Random Forest (RF)^20^. Another research used routine clinical data to develop a model to predict the risk of carotid plaque progression in patients with asymptomatic carotid stenosis. They found that logistic regression (LR) could provide the best predictive ability of AUC at 0.809^21^. In metabolomics biomarker discovery, seven lipoprotein-focused metabolites have been identified by the top features of lasso LR and random forest machine learning models. Leda et al*.* found that for metabolic profiles, logistic regression achieved a maximum accuracy of 0.8^22^. Song et al*.* even developed a novel multi-metabolite predictive model to predict response to statin therapy in patients with atherosclerosis. They identified RA-specific abnormalities in remitted patients after PCI dominated by alternations in lipid biochemical pathways, including sphingolipid, phospholipid, eicosanoid, and fatty acid oxidation. The AUC and accuracy were up to 0.89 and 0.90, respectively^23^.

Although these studies have demonstrated a good performance in biomarker discovery and disease prediction, their limited AUC and accuracy hinder their scalability for clinical use. To further improve the performance, we propose a new method integrates multiple ML algorithms and feature selection method to handle multidimensional data. Different strengths and weaknesses of the algorithms are considered to find the best fit model, and the feature selection method identifies informative and relevant features to improve model generalization and reduce overfitting. Notably, we taken into consideration the importance of shared features for disease across different models. These features with strong predictive power for disease can be selected as candidate biomarkers for further research.

We found (1) The combination of clinical factor and metabolite profile provides stability to data set shifts; (2) with feature selection method we improved the model performance from an AUC of 0.89 to 0.92; (3) the shared features had predictive power equivalent to 67 features, suggesting their clinical importance in identifying patients with LAA. In this study, we attempted to develop a new biomarker discovery method which may help identify LAA less costly and more efficient.

## Methods

### Participants and study design

From 2010 to 2015, consecutive ischemic stroke patients with extracranial LAA were recruited according to the following inclusion criteria: (1) cerebral angiography including digital subtraction, magnetic resonance or computed tomographic angiogram exhibiting evidence of the extracranial common and internal carotid artery having ≥ 50% diameter stenosis according to NASCET criteria^24^; (2) stable neurological condition during blood sample collection; (3) no acute illness, such as infection or inflammation, at the time of blood sample collection; and (4) a modified Rankin Scale score of less than 3. Normal controls were recruited from the neurology outpatient department. Normal controls were defined as those with (1) no history of stroke and coronary artery disease, (2) brain magnetic resonance or computed tomographic angiogram exhibiting < 50% diameter stenosis at bilateral intracranial and extracranial carotid arteries, and (3) no acute illness during blood sample collection.

The exclusion criteria were (1) exhibiting systemic diseases, such as hypothyroidism or hyperthyroidism, decompensated liver cirrhosis, acute kidney injury, or systemic lupus erythematosus and (2) having cancer and other serious illnesses during recruitment. This study was approved by the Institutional Review Board of Linkou Chang Gung Memorial Hospital (revised approval numbers: 201506352B0C501 and 202000552B0C601). All the participants signed informed consent forms before being recruited into this study.

Venous blood samples and clinical profiles were collected at recruitment of normal controls and LAA patients in stationary condition. Blood for metabolomics analysis was stored in sodium citrate tubes and centrifuged (10 min, 3000 rpm at 4 °C) within an hour after collection. Plasma was aliquoted into separate polypropylene tubes and stored at − 80 °C freezer. The measurement of metabolites was done following our previous method (Lin CN et al., 2021) using the targeted Absolute IDQ®p180 kit (Biocrates Life Science, AG, Innsbruck, Austria) which can quantify 194 endogenous metabolites from 5 classes of compound. The assay was performed by using a Waters Acquity Xevo TQ-S instrument (Waters, Milford, MA, USA). The level of metabolite was obtained by using the Biocrates®® MetIDQ™ software.

### Data preprocessing and parameters of machine learning models

**Figure 1:**
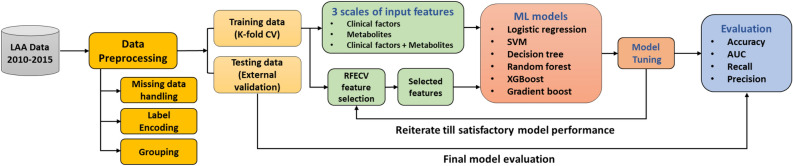
The flowchart of ML models used for the prediction of LAA. *AUC* area under the receiver operating characteristic curve; *CV* cross-validation, *LAA* large-artery atherosclerosis; *ML* machine learning; *RFECV* recursive feature elimination with cross-validation; *SVM* support vector machine; *XGBoost* extreme gradient boosting.

The workflow of this study is shown in Fig. 1. The data preprocessing steps involved missing data handling, label encoding, and participant grouping. Open-source specialized packages for Python, including Pandas^24,25^, NumPy^26^, scikit-learn^27^, Matplotlib^28^, Seaborn^29^, TableOne^30^, and SciPy^31^, were applied. We used the mean imputation method of the Useful package^32^ in R to obtain the missing values for each variable. After converting the categorical variables into dummy variables, we used 80% of the data set for model training/validation (tenfold cross-validation training set, n = 287) and the remaining 20% for performance testing (external validation set, n = 72). Three scales of input features including clinical factors, metabolites and clinical factors + metabolites were adopted using six machine learning models: logistic regression (LR), support vector machine (SVM), decision tree, random forest (RF), extreme gradient boosting (XGBoost), and gradient boosting (Supplementary Methods).

LR is a supervised learning technique used to address classification issues and determine the likelihood of a binary (yes/no) occurrence. Whatever the variable is dichotomous or categorical, the logistic function use a S-shaped curve to transform data into a value between 0 and 1 for classification issues^33^.

SVM was first proposed by Corinna et al.^34^. Processing nonlinear, small-sample, and high-dimensional pattern recognition problems with SVM provides various benefits. It offers a great generalization ability for unknown samples because the partitioning hyperplane may ensure that the extreme solution is a global optimal solution rather than a local minimal value and has a solid theoretical foundation^35^.

Decision tree algorithm is a common type of machine learning algorithm in which decisions are made according to a tree structure. A decision tree typically has a root node, a number of internal nodes, and a number of leaf nodes. The root node includes all of the samples, and each node's samples are separated into subnodes based on the outcomes of an attribute test. The sequence of decision tests corresponds to the route from the root node to the last leaf node^35,36^.

RF is an extension of the bagging method^37^, which is a typical ensemble learning method. Bagging often entails processing chores using a straightforward voting system. A decision tree algorithm serves as the foundation learner for RF, and during decision tree training, random attribute selection is included. For a variety of real-world data, RF offers reliable performance, and is easily understood^35^. It has shown good performance in applications like disease prediction, gene selection, and picture recognition^38–40^.

XGBoost is a novel gradient boosting ensemble learning method. In this method, machine learning is implemented under the gradient boosting framework with high efficiency, flexibility, and portability^41^. Tree boosting is an efficient and widely used machine learning method that is a type of boosted ensemble learning^42^. The second-order Taylor expansion of the loss function is used by the XGBoost model, and a regularization function is added to this expansion to strike a compromise between the model's complexity and loss function reduction. This approach attempts to avoid overfitting to some extent by looking for the overall ideal solution^35^. The gradient tree boosting algorithm used by XGBoost is to increase its speed and accuracy.

Gradient boosting is a fast and accurate machine-learning-based prediction method that is particularly well suited for large and complicated datasets. Gradient boosting redefines boosting as a numerical optimization problem with the objective of minimizing the loss function by incorporating a weak learner via gradient descent. In order to reduce the overall error of the strong learner, the contribution of each weak learner to the final prediction is based on a gradient optimization procedure. Gradient boosting focuses on existing underperforming learners^43^.

In the models of this study, we set the maximum number of iterations as 3,000 and added a penalty term (L2) to the loss function in our LR model. For our SVM model, the radial basis function was used as the kernel function, the regularization parameter (C) was 1.0, and the class weight was set as “balanced.” For the decision tree model, the maximum depth of a tree was 6, it was determined through optimization procedures and based on previous studies. For the RF and gradient boosting algorithms, default parameter settings were used. For the XGBoost algorithm, we used the tree construction algorithm (tree_method) as “hist”, and nodes with the highest loss change were added to the tree (grow_policy).

### Feature selection through recursive feature elimination with cross-validation

**Figure 2:**
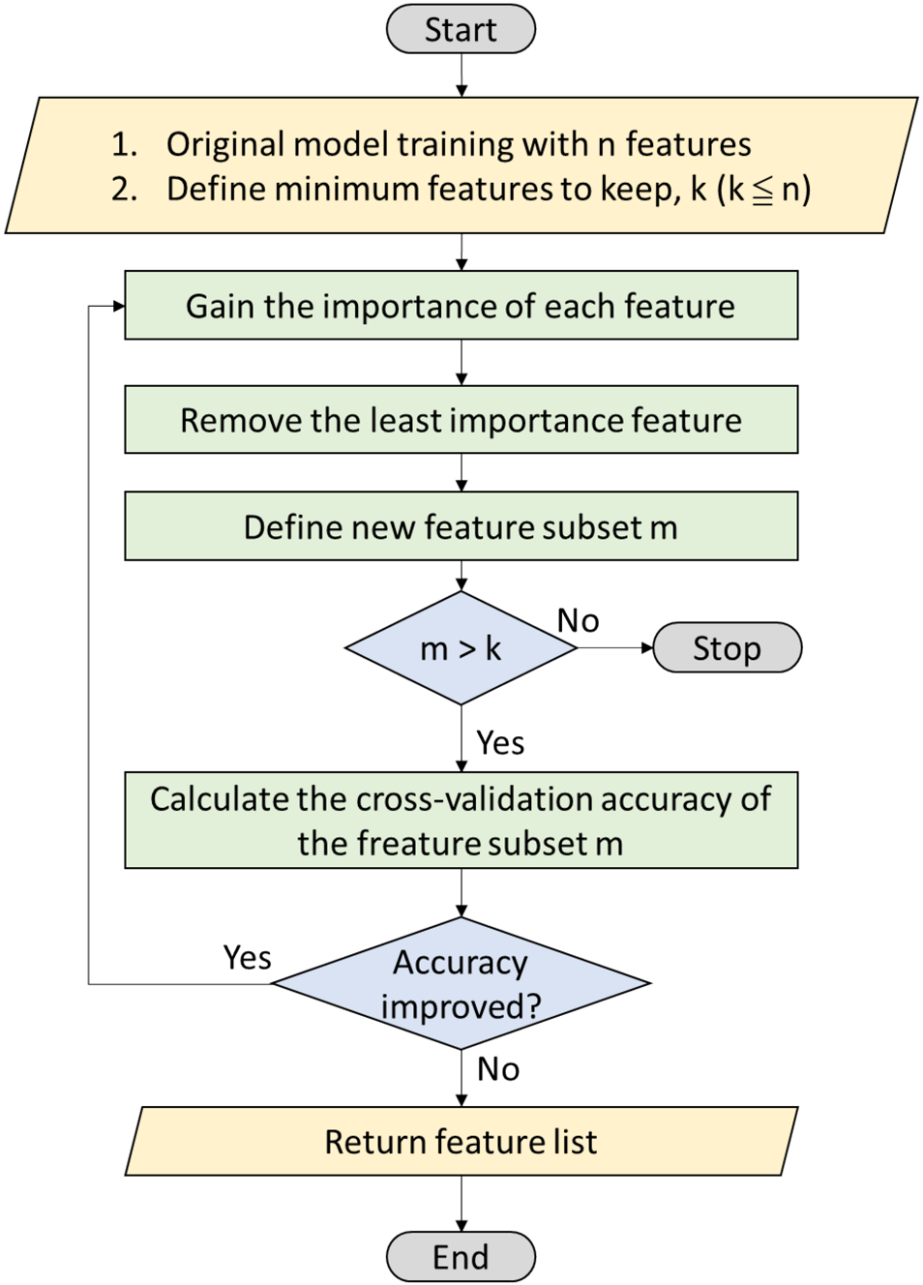
The flowchart of recursive feature elimination with cross-validation (RFECV) method.

For reduction of the number of input variables of the machine learning models, we used the recursive feature elimination (RFE) with cross-validation (RFECV) method to identify the important features in this study (Fig. 2). The RFECV method has received considerable research attention because of its robustness^44^. RFE is a greedy algorithm based on the packing model. The RFECV algorithm starts from a complete feature set, and its performance metric is the prediction accuracy or area under the receiver operating characteristic curve (AUC) of the classifier. At the end of an iteration, the least relevant features are eliminated. The most relevant features are then sorted and extracted. RFE involves the extraction of feature subsets according to the feature ranking table generated on the basis of the aforementioned evaluation metrics^35^.

### Model evaluation metrics

Baseline metabolite levels and clinical factors were presented in terms of mean ± standard deviation. Categorical variables were expressed as absolute and percentage frequencies. The Python 3.7 software package and scikit-learn toolkit were adopted, and the default settings were applied to train with the LR, SVM, decision tree, RF, XGBoost, and gradient boost algorithms.

We used the four metrics including accuracy, AUC, recall, and precision to evaluate the performance of the machine learning models.

$${\text{Accuracy}} = \frac{TP + TN}{{TP + FN + TN + FP}}$$

$${\text{Recall }} = \frac{TP}{{TP + FN}}$$

$${\text{Precision }} = \frac{TP}{{TP + FP}}$$

where TP denotes true positives, FP represents false positives, TN denotes true negatives, and FN refers to false negatives.

The input data was split into a training set and an external validation set, following an 8:2 ratio. Subsequently, the training set was divided into *k* equal parts for *k*-fold internal cross-validation. During each of the *k* iterations, one part of the training set was designated for internal validation, while the remaining *k* − 1 parts were employed for training the models. This approach facilitated thorough evaluation and model training using the training sets. Lastly, the performance of the models was evaluated using the external validation set. This procedure was repeated until each of the *k* subsets had been served as the validation set. The average of the *k* performance measurements was the cross-validated performance^45^. In this study, we conducted internal stratified tenfold cross-validation in the training set to estimate the performance of the models^46^. And the final performance of the models was evaluated using the external validation set. The RFECV algorithm was used to determine the contributions of features to the predictions classified into the LAA and “control” categories.

### Mean absolute difference (MAD)

The mean absolute difference (MAD) is a statistical measure used to quantify the average discrepancy between individual data points and a reference value^47^. It is calculated by taking the absolute difference between each data point and the reference value, then computing the average of these absolute differences. MAD provides valuable insights into the dispersion or variability of a dataset, allowing researchers to assess the magnitude of differences from a central reference point.

### Ethics statement

All methods described in this study were carried out in accordance with relevant guidelines and regulations. The studies involving human participants were reviewed and approved by the Institutional Review Board of Linkou Chang Gung Memorial Hospital (revised approval numbers: 201506352B0C501 and 202000552B0C601) and informed consent was obtained from all participants prior to their inclusion in the study. The study also adhered to the principles outlined in the Declaration of Helsinki and the International Conference on Harmonization-Good Clinical Practice (ICH-GCP) guidelines.

## Results

### Patient population and demographics

There were 359 people who participated in the study, with 176 of them were LAA, and the remaining 183 were normal control (Table 1 and Supplementary Fig. S1). The mean age of the LAA cohort was 64 years (range: 58–69 years), while the control cohort was 61 years (range: 56–66 years). The two groups of individuals who participated in the study did not differ in sex, family history of stroke, chronic kidney disease, and most anthropometric measures. However, there were significant differences between the two groups in hypertension, diabetes mellitus, and usage of long-term medication (*p* < 0.001). Compared with the normal controls, the patients with LAA were almost twice as likely as normal controls to exhibit unhealthy lifestyle behaviors such as smoking (40.4% vs. 73.3%, *p* < 0.001) and alcohol usage (24.4% vs. 42.0%, *p* < 0.001). Moreover, the patients with LAA had higher levels of homocysteine and creatinine but lower levels of high-density/low-density lipoprotein and total cholesterol (all *p* < 0.01) than the normal controls.

**Table 1:** Clinical factors of the 359 study participants.

Variable	N	LAA, N = 176^1^	Control, N = 183^1^	*p*-value^2^
Age	359	64 (58, 69)	61 (56, 66)	0.001
Male		163 (92.6%)	166 (90.7%)	0.500
Risk factor				
Hypertension (HTN)	359	132 (75.0%)	83 (45.4%)	< 0.001
Diabetes mellitus (DM)	359	56 (31.8%)	11 (6.0%)	< 0.001
Smoking	359	129 (73.3%)	74 (40.4%)	< 0.001
Alcohol	359	74 (42.0%)	44 (24.4%)	< 0.001
Family history of stroke	359	65 (36.9%)	62 (33.9%)	0.500
Chronic kidney disease	359	1 (0.57%)	1 (0.55%)	> 0.900
Body height (cm)	349	163 (160, 168)	165 (160, 169)	0.300
Body weight (kg)	349	65 (60, 71)	68 (62, 75)	0.001
Body mass index (BMI)	349	24.39 (22.85, 26.35)	25.55 (23.14, 27.57)	0.003
Waistline size (cm)	325	84 (79, 91)	85 (79, 90)	0.600
Hip size (cm)	325	90 (85, 95)	92 (86, 96)	0.021
Systolic blood pressure (mm Hg)	345	134 (118, 148)	132 (120, 145)	0.400
Diastolic blood pressure (mm Hg)	345	75 (66, 84)	79 (73, 87)	< 0.001
Mean blood pressure (mm Hg)	345	94 (85, 105)	96 (89, 106)	0.068
Heart rate (bpm)	345	73 (65, 82)	74 (67, 84)	0.200
Blood test				
Homocysteine (mg/dL)	327	11.20 (9.50, 13.0)	10.10 (8.47, 11.90)	< 0.001
Glucose (fasting, mg/dL)	331	98 (89.0, 114.0)	97 (90, 106)	0.600
High sensitive C-reactive protein (mg/dL)	327	1.6 (0.8, 4.0)	1.2 (0.6, 2.6)	0.006
High-density lipoprotein cholesterol (mg/dL)	358	39 (34, 46)	47 (40, 55)	< 0.001
Low-density lipoprotein cholesterol (mg/dL)	358	106 (77, 128)	114 (91, 135)	0.001
Triglyceride (mg/dL)	358	117 (87, 160)	104 (76, 166)	0.300
Total cholesterol (mg/dL)	358	170 (146, 192)	188 (164, 214)	< 0.001
Uric Acid (mg/dL)	357	6.40 (5.15, 7.40)	6.05 (5.40, 7.07)	0.500
Creatinine (mg/dL)	342	0.95 (0.80, 1.20)	0.89 (0.78, 0.99)	< 0.001
Medications used within 3 months before blood sample collection				
Anti-hypertensive	359	88 (50.0%)	25 (13.6%)	< 0.001
Anti-diabetic	359	34 (19.3%)	1 (0.55%)	< 0.001
Anti-lipid	359	108 (61.4%)	27 (14.8%)	< 0.001

^1^Numerical data are presented as medians (interquartile range), and categorical data are presented in terms of N (%).

^2^The Wilcoxon rank sum test is used for analyzing continuous variables; Pearson’s chi-squared test is used for examining categorical variables for which expected cell counts are ≥ 5; and Fisher's exact test is used for investigating categorical variables for which expected cell count is < 5.

*LAA* large-artery atherosclerosis.

In the analysis of serum metabolites, significant differences were observed between the two groups of 68 out of the 164 analyzed metabolites (41.5%; Table 2). Patients with LAA exhibited lower levels of 34 phosphatidylcholines (PCs, 50.0% in 68) than the normal controls, whereas higher levels of amino acids (6 out of 21; 28.6%), biogenic amines (two out of six; 33.3%), lysoPCs (1 out of 12; 8.3%), and other markers (three out of seven; 42.9%) were observed in the LAA group (all; *p* < 0.05). Additionally, 17 acylcarnitines showed differences between the two groups, with 13 acylcarnitines exhibiting lower levels and 4 showing higher levels in the LAA group (*p* < 0.05). Of the 11 sphingomyelins examined, only SMOHC141 showed a significant difference between the two groups (*p* = 0.006). The examined metabolites are presented in Supplementary Table 1 and Fig. S2.

**Table 2:** Comparison of serum metabolites between patients with large-artery atherosclerosis (LAA) and normal controls.

Variable	N	LAA, N = 176^1^	Control, N = 183^1^	*p*-value^2^	Variable	N	LAA, N = 176^1^	Control, N = 183^1^	*p*-value^2^
**Acylcarnitines**					**Phosphatidylcholines**				
C10	359	0.14 (0.11, 0.22)	0.20 (0.15, 0.26)	< 0.001	PCaaC281	359	0.92 (0.69, 1.32)	1.02 (0.82, 1.33)	0.012
C101	305	0.36 (0.30, 0.42)	0.40 (0.35, 0.46)	< 0.001	PCaaC323	359	0.16 (0.14, 0.19)	0.19 (0.16, 0.22)	< 0.001
C12	359	0.074 (0.060, 0.097)	0.089 (0.072, 0.110)	< 0.001	PCaaC343	359	5.40 (4.27, 7.00)	6.05 (4.70, 7.74)	0.014
C14	305	0.030 (0.026, 0.038)	0.032 (0.027, 0.038)	0.045	PCaaC344	359	0.51 (0.38, 0.69)	0.60 (0.46, 0.75)	0.001
C121	359	0.28 (0.20, 0.38)	0.30 (0.23, 0.38)	0.032	PCaaC360	359	2.22 (1.55, 2.91)	2.35 (1.72, 3.37)	0.032
C141	359	0.055 (0.047, 0.069)	0.068 (0.056, 0.080)	< 0.001	PCaaC361	359	24 (18, 32)	27 (21, 35)	0.013
C141OH	305	0.012 (0.010, 0.016)	0.014 (0.011, 0.017)	0.005	PCaaC364	359	96 (85, 110)	91 (79, 103)	0.024
C142	359	0.028 (0.018, 0.047)	0.035 (0.024, 0.051)	0.003	PCaaC365	359	8 (6, 13)	10 (7, 15)	0.046
C161	359	0.019 (0.014, 0.026)	0.022 (0.016, 0.029)	0.018	PCaaC366	359	0.28 (0.19, 0.37)	0.34 (0.24, 0.47)	< 0.001
C18	305	0.032 (0.026, 0.042)	0.036 (0.030, 0.043)	0.004	PCaaC380	359	2.11 (1.70, 2.56)	2.32 (1.90, 2.91)	< 0.001
C181OH	305	0.0070 (0.0060, 0.0083)	0.0070 (0.0070, 0.0090)	0.019	PCaaC402	359	0.26 (0.20, 0.32)	0.28 (0.22, 0.36)	0.002
C3	359	0.33 (0.24, 0.42)	0.29 (0.23, 0.38)	0.039	PCaaC422	359	0.21 (0.17, 0.26)	0.25 (0.21, 0.31)	< 0.001
C4	359	0.20 (0.16, 0.28)	0.16 (0.13, 0.20)	< 0.001	PCaaC424	359	0.16 (0.13, 0.18)	0.16 (0.14, 0.19)	0.015
C5	359	0.12 (0.09, 0.16)	0.10 (0.08, 0.13)	< 0.001	PCaaC425	359	0.21 (0.17, 0.26)	0.22 (0.18, 0.30)	0.023
C5MDC	305	0.025 (0.022, 0.030)	0.024 (0.020, 0.029)	0.038	PCaaC426	359	0.26 (0.21, 0.32)	0.28 (0.23, 0.36)	0.020
C51DC	305	0.01 (0.01, 0.02)	0.02 (0.01, 0.43)	0.019	PCaeC300	359	0.12 (0.10, 0.16)	0.15 (0.12, 0.17)	< 0.001
C7DC	359	0.026 (0.017, 0.039)	0.032 (0.025, 0.044)	< 0.001	PCaeC302	359	0.040 (0.033, 0.047)	0.046 (0.039, 0.054)	< 0.001
C8	359	0.16 (0.13, 0.21)	0.20 (0.15, 0.25)	< 0.001	PCaeC321	359	1.11 (0.87, 1.38)	1.24 (1.04, 1.54)	< 0.001
C9	305	0.020 (0.016, 0.026)	0.023 (0.019, 0.028)	0.002	PCaeC322	359	0.29 (0.23, 0.36)	0.35 (0.28, 0.43)	< 0.001
**Amino acids**					PCaeC340	359	0.56 (0.44, 0.67)	0.63 (0.53, 0.74)	< 0.001
Aspartic acid	359	3.03 (2.08, 5.20)	2.30 (1.40, 3.70)	< 0.001	PCaeC342	359	4.81 (3.86, 6.18)	5.97 (4.89, 7.20)	< 0.001
Citrulline	359	26 (20, 35)	24 (18, 29)	0.003	PCaeC343	359	3.34 (2.73, 4.22)	4.28 (3.42, 5.22)	< 0.001
Glutamic acid	359	53 (38, 74)	43 (33, 60)	< 0.001	PCaeC360	359	0.46 (0.38, 0.55)	0.51 (0.42, 0.60)	< 0.001
Isoleucine	359	76 (61, 89)	69 (58, 79)	0.002	PCaeC362	359	6.25 (5.28, 7.51)	7.11 (6.16, 8.55)	< 0.001
Methionine	359	21 (18, 27)	23 (20, 27)	0.018	PCaeC363	359	3.09 (2.56, 3.82)	3.67 (3.08, 4.34)	< 0.001
Ornithine	359	68 (54, 102)	65 (47, 88)	0.028	PCaeC364	359	7.97 (6.41, 9.73)	8.88 (7.15, 10.85)	0.005
Phenylalanine	359	66 (57, 77)	62 (57, 69)	0.011	PCaeC365	359	5.84 (4.81, 7.02)	6.71 (5.16, 7.93)	< 0.001
Proline	359	184 (141, 231)	150 (126, 188)	< 0.001	PCaeC380	359	0.78 (0.61, 0.95)	0.92 (0.71, 1.13)	< 0.001
Tryptophan	359	45 (38, 54)	50 (44, 57)	< 0.001	PCaeC382	359	0.78 (0.54, 1.13)	0.91 (0.65, 1.22)	0.009
**Biogenic amines**					PCaeC385	359	7.68 (6.18, 8.80)	7.91 (6.93, 9.41)	0.045
Kynurenine	359	1.90 (1.60, 2.32)	1.70 (1.46, 2.08)	< 0.001	PCaeC386	359	3.46 (2.91, 4.18)	4.08 (3.24, 5.04)	< 0.001
SDMA	359	0.50 (0.42, 0.62)	0.47 (0.40, 0.51)	< 0.001	PCaeC401	359	0.70 (0.51, 0.94)	0.78 (0.60, 0.97)	0.007
**Sphingomyelins**					PCaeC406	359	2.54 (2.13, 3.05)	2.68 (2.26, 3.26)	0.025
SMOHC141	359	3.54 (3.05, 4.40)	4.11 (3.24, 4.96)	0.006	PCaeC422	359	0.31 (0.24, 0.39)	0.33 (0.28, 0.42)	0.011
**Others**					PCaeC423	359	0.51 (0.39, 0.63)	0.54 (0.46, 0.68)	0.006
ADMA	359	0.40 (0.30, 0.50)	0.34 (0.30, 0.40)	0.005	**Lysophosphatidylcholines**				
Creatinine_MS	359	88 (70, 106)	81 (65, 95)	0.010	lysoPCaC204	359	4.55 (3.14, 6.37)	3.94 (3.01, 5.27)	0.018
SMOHC241	359	1.29 (1.09, 1.49)	1.37 (1.06, 1.70)	0.031					

^1^Numerical data are presented as medians (interquartile range), and categorical data are presented in terms of N (%).

^2^The Wilcoxon rank-sum test is used for analyzing continuous variables; Pearson's chi-squared test is used for examining categorical variables for which expected cell counts are ≥ 5; and Fisher’s exact test is used for investigating categorical variables for which expected cell count is < 5.

### Performance of the adopted models in predicting LAA using three scales of input features

The performance of the 6 predictive models is presented in Table 3, using the 10-fold cross validation. After training, all of these models exhibited high AUC values but low precision, recall, and accuracy. When the training was performed on clinical factors, metabolites and clinical factors + metabolites, the mean performances in clinical factors were accuracy: 0.76 ± 0.18, AUC: 0.84 ± 0.15, recall: 0.75 ± 0.28, precision: 0.75 ± 0.20; those in metabolites were accuracy: 0.70 ± 0.13, AUC: 0.76 ± 0.12, recall: 0.68 ± 0.20, precision: 0.69 ± 0.16; those in clinical factors + metabolites were accuracy: 0.77± 0.13, AUC: 0.83 ± 0.12, recall: 0.74 ± 0.23, precision: 0.77 ± 0.16. We found that the models trained with clinical factors had similar performance to models trained with clinical factors + metabolites in predicting LAA, but the best fit algorithm was different for each input feature scale. When considering only clinical factors for training the models, the RF model exhibited the best LAA prediction performance in all the 4 metrics (AUC: 0.90 ± 0.14, accuracy: 0.82 ± 0.17, recall: 0.81 ± 0.26, and precision: 0.82 ± 0.21). When considering only metabolites factors, the LR model exhibited the best AUC value of 0.81 ± 0.10 with accuracy: 0.73 ± 0.12, recall: 0.72 ± 0.12, and precision: 0.72 ± 0.17. When combining both clinical factors and metabolites factors, the LR model exhibited the best AUC value of 0.89 ± 0.12 with accuracy: 0.78 ± 0.15, recall: 0.77 ± 0.26, and precision: 0.77 ± 0.17.

**Table 3:** Performance of the six predictive models using 3 scales of input features using the tenfold cross validation.

Model	Features	Accuracy	AUC	Recall	Precision
Logistic regression	Clinical factors	0.78 (± 0.17)	0.88 (± 0.12)	0.74 (± 0.27)	0.79 (± 0.21)
SVM	Clinical factors	0.77 (± 0.19)	0.87 (± 0.15)	0.77 (± 0.28)	0.75 (± 0.19)
Decision tree	Clinical factors	0.68 (± 0.23)	0.71 (± 0.23)	0.68 (± 0.33)	0.65 (± 0.26)
**Random forest**	**Clinical factors**	**0.82 (± 0.17)**	0.90 (± 0.14)*	**0.81 (± 0.26)**	**0.82 (± 0.21)**
XGBoost	Clinical factors	0.77 (± 0.13)	0.87 (± 0.11)	0.77 (± 0.27)	0.76 (± 0.14)
Gradient boost	Clinical factors	0.77 (± 0.19)	0.86 (± 0.16)	0.76 (± 0.29)	0.75 (± 0.19)
**Mean**	Clinical factors	0.76 (± 0.18)	0.84 (± 0.15)	0.75 (± 0.28)	0.75 (± 0.20)
**Logistic regression**	**Metabolites**	**0.73 (± 0.12)**	0.81 (± 0.10)*	**0.72 (± 0.12)**	**0.72 (± 0.17)**
SVM	Metabolites	0.72 (± 0.13)	0.80 (± 0.13)	0.75 (± 0.11)	0.70 (± 0.19)
Decision tree	Metabolites	0.61 (± 0.15)	0.60 (± 0.18)	0.55 (± 0.33)	0.59 (± 0.16)
Random forest	Metabolites	0.71 (± 0.15)	0.79 (± 0.13)	0.69 (± 0.22)	0.72 (± 0.22)
XGBoost	Metabolites	0.74 (± 0.13)	0.80 (± 0.14)	0.69 (± 0.26)	0.74 (± 0.12)
Gradient boost	Metabolites	0.71 (± 0.10)	0.79 (± 0.09)	0.68 (± 0.19)	0.70 (± 0.11)
**Mean**	Metabolites	0.70 (± 0.13)	0.76 (± 0.12)	0.68 (± 0.20)	0.69 (± 0.16)
**Logistic regression**	**Clinical factors + Metabolites**	**0.78 (± 0.15)**	0.89 (± 0.12)*	**0.77 (± 0.26)**	**0.77 (± 0.17)**
SVM	Clinical factors** + **Metabolites	0.77 (± 0.16)	0.85 (± 0.13)	0.77 (± 0.20)	0.76 (± 0.22)
Decision tree	Clinical factors** + **Metabolites	0.71 (± 0.08)	0.68 (± 0.16)	0.67 (± 0.17)	0.71 (± 0.13)
Random forest	Clinical factors** + **Metabolites	0.78 (± 0.18)	0.86 (± 0.12)	0.76 (± 0.22)	0.78 (± 0.21)
XGBoost	Clinical factors** + **Metabolites	0.80 (± 0.11)	0.87 (± 0.11)	0.75 (± 0.26)	0.81 (± 0.12)
Gradient boost	Clinical factors** + **Metabolites	0.81 (± 0.15)	0.88 (± 0.12)	0.77 (± 0.27)	0.82 (± 0.15)
**Mean**	Clinical factors + Metabolites	0.77 (± 0.13)	0.83 (± 0.12)	0.74 (± 0.23)	0.77 (± 0.16)

*Represents the highest AUC value among the six models when different feature selection methods are used. *AUC* area under the receiver operating characteristic curve; *SVM* support vector machine; *XGBoost* extreme gradient boosting.

Significant values are in [bold].

**Figure 3:**
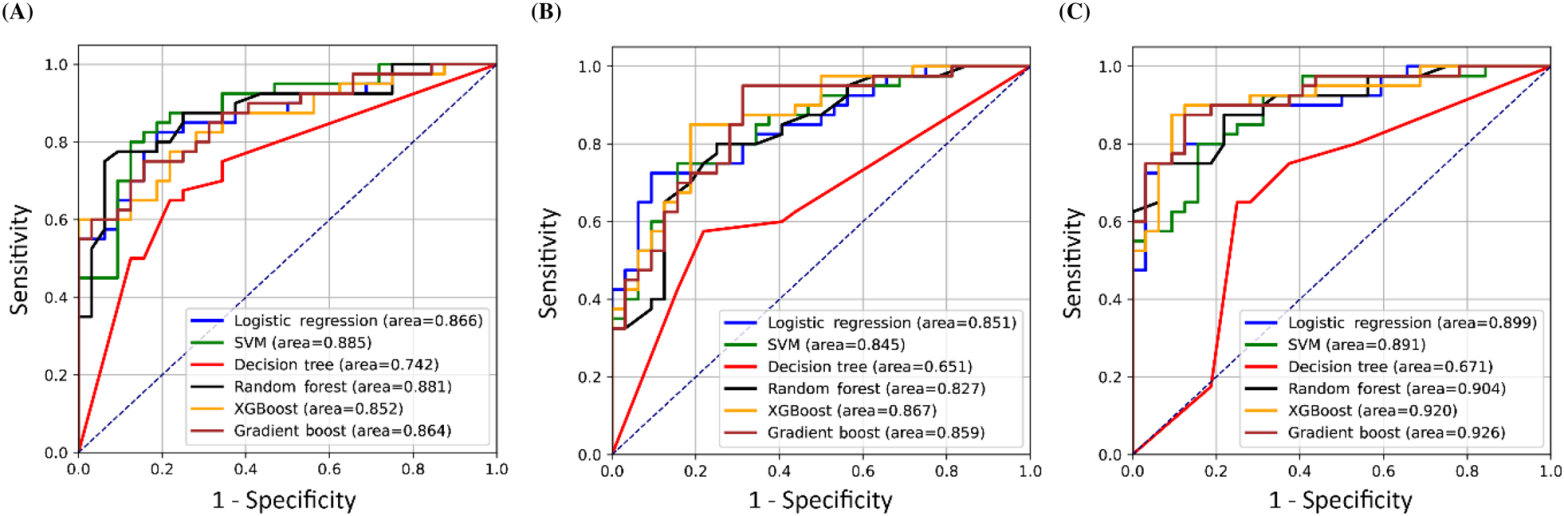
Receiver operating characteristic curves for the 6 machine learning models evaluated with the external validation set using 3 scales of input features: (**A**) clinical factors, (**B**) metabolites, and (**C**) combination of clinical factors and metabolites. *SVM* support vector machine, *XGBoost* extreme gradient boosting.

To test the robustness, we evaluated the model performance by the AUCs within the external validation set. The mean AUC for the 6 models tested by clinical factors, metabolites and clinical factors + metabolites were 0.84 ± 0.04, 0.81 ± 0.07, and 0.86 ± 0.08, respectively (Fig. 3). In models tested by clinical factors, the SVM exhibited the best AUC value of 0.88 [Fig. 3A]. However, in models tested by metabolites, all AUC values decreased, especially the decision tree model [AUC: 0.65, Fig. 3B]. The XGBoost exhibited the best AUC value of 0.86 [Fig. 3B]. In models tested by clinical factors + metabolites, the AUC of the decision tree model increased to 0.67, and the others also increased to over 0.89 [Fig. 3C]. Gradient boost model exhibited the best AUC value of 0.92 [Fig. 3C]. The mean absolute difference (MAD) between training and testing dataset were then calculated (Table 4). The MAD of logistic regression model was 0.02, which was the lowest among the 6 models. Indicating, LR model consistently exhibited the best or second-best performance for different input scales.

**Table 4:** Comparison of the area under the receiver operating characteristic curve between the training set and external validation set.

	Clinical factors	Metabolites	Clinical factors + Metabolites	MAD	Rank
Logistic regression	0.01	− 0.04	− 0.01	0.02	1
SVM	− 0.02	− 0.05	− 0.04	0.04	4
Decision tree	− 0.03	− 0.05	0.01	0.03	2
Random forest	0.02	− 0.04	− 0.04	0.03	2
XGBoost	0.02	− 0.07	− 0.05	0.05	6
Gradient boost	0	− 0.07	− 0.05	0.04	4

*MAD* mean absolute difference; *SVM* support vector machine; *XGBoost* extreme gradient boosting.

### Feature selection by using the RFECV method

**Figure 4:**
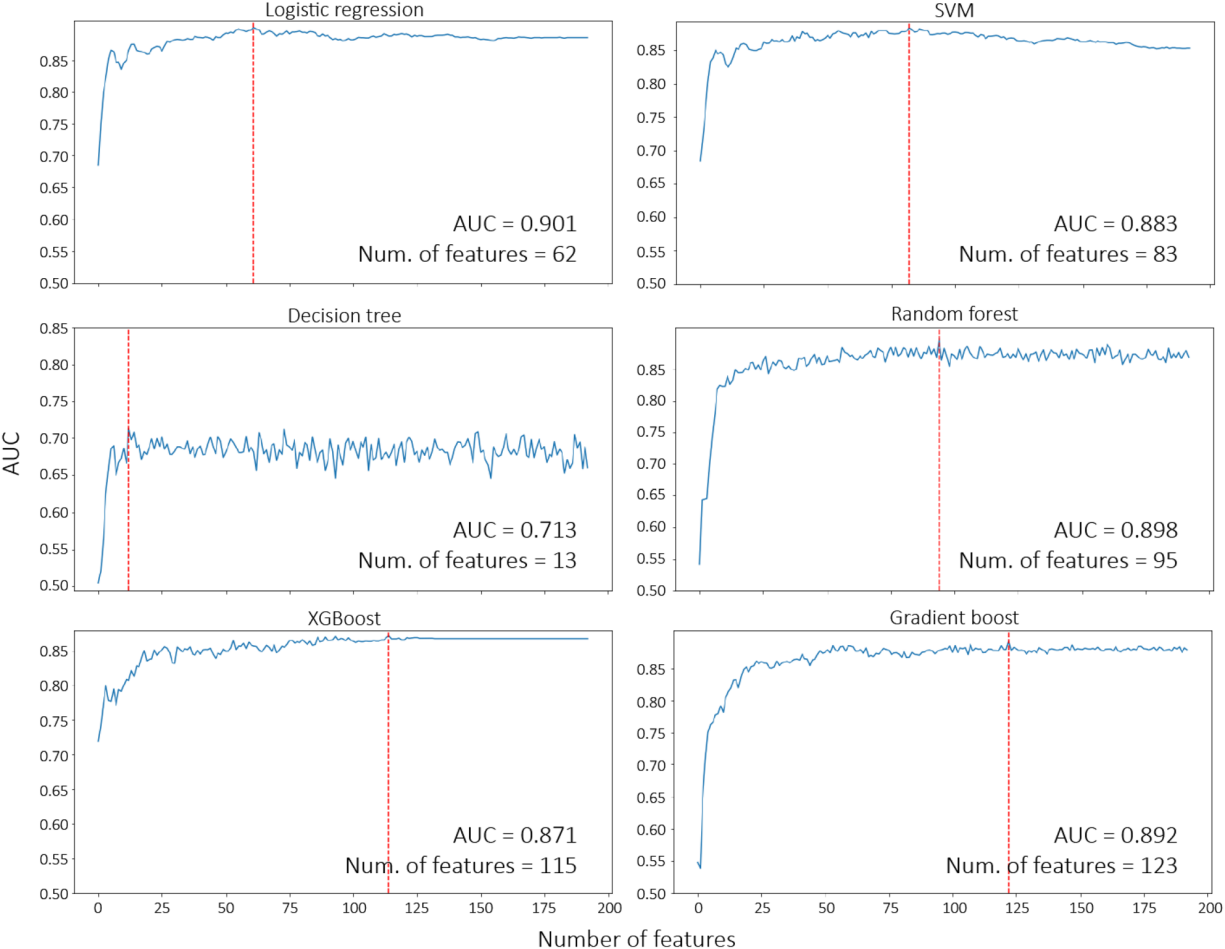
RFECV curves for the 6 adopted ML models. The red dot-line represents the number of features required to attain the highest AUC value. *AUC* area under the receiver operating characteristic curve; *ML* machine learning; *RFECV* recursive feature elimination with cross-validation; *SVM* support vector machine; *XGBoost* extreme gradient boosting.

Tables 3 and 4 show using both clinical factors and metabolites as features provided the best AUC and robustness over the 6 models. Figure 4 illustrates the selection process to obtain the best subset of features from clinical factors + metabolites (193 features) in each model (Supplementary Table 2). The results indicated that five models achieved an AUC value over 0.87 when using the RFECV method (Fig. 4). Only the decision tree model exhibited an AUC value of 0.71. Among the 6 models, the LR model used the least number of features but achieved the best performance (AUC = 0.90).

**Figure 5:**
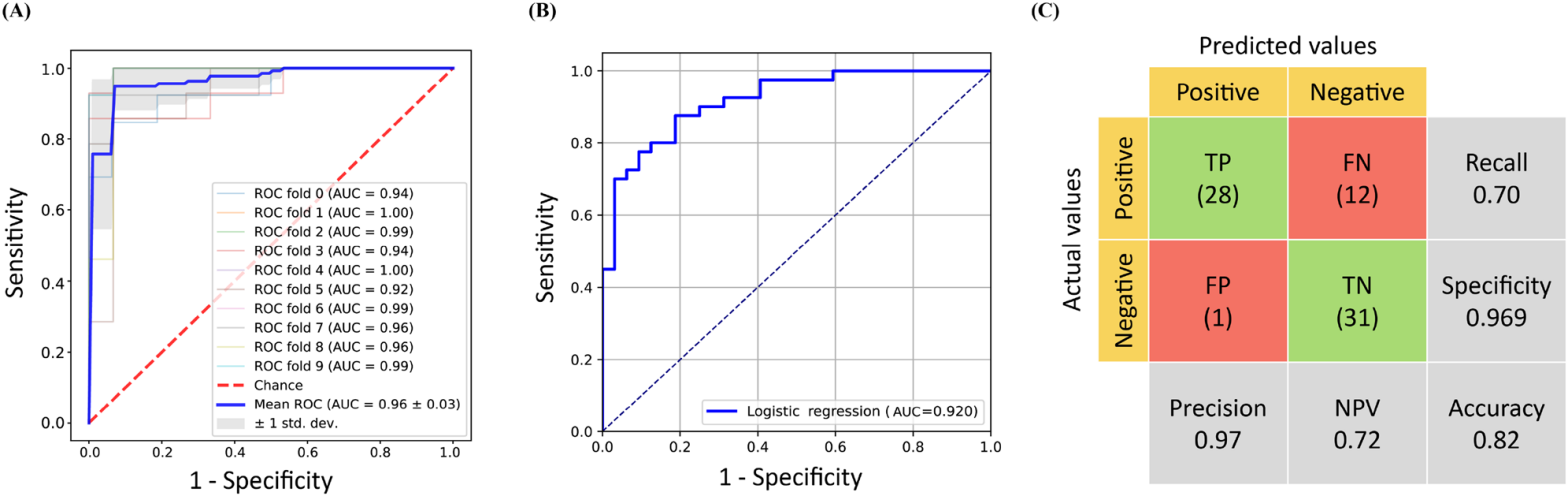
Feature selection using the RFECV method for the LR algorithm (62 features): (**A**) receiver operating characteristic curves for tenfold cross-validation on the training set, (**B**) receiver operating characteristic curves on the external validation set, and (**C**) confusion matrix for the external validation set. *FN* false negative; *FP* false positive; *LR* logistic regression; *NPV* negative predictive value; *RFECV* recursive feature elimination with cross-validation; *TN* true negative; *TP* true positive.

Since the AUC score did not increase substantially when more than 62 features were selected, 62 features were selected to train a new LR model. These features comprised 15 clinical factors and 47 metabolites (Supplementary Table 3). A tenfold cross-validation was performed on the training set to estimate the generalization capability of the LR model. Similar AUC values were obtained for each part of the training set, indicating that no overfitting occurred [Fig. 5A]. The mean AUC value of the LR model was 0.96 ± 0.03 which was better than the other models trained with different original inputs and methods. When the external validation set was used, an AUC value of 0.92 was obtained for the LR model [Fig. 5B] with accuracy = 0.82 [Fig. 5C], with six patients being misclassified as belonging to the normal cohort and six members of the normal cohort being misclassified as patients with LAA.

### Shared features could improve the performance of the 6 models

**Figure 6:**
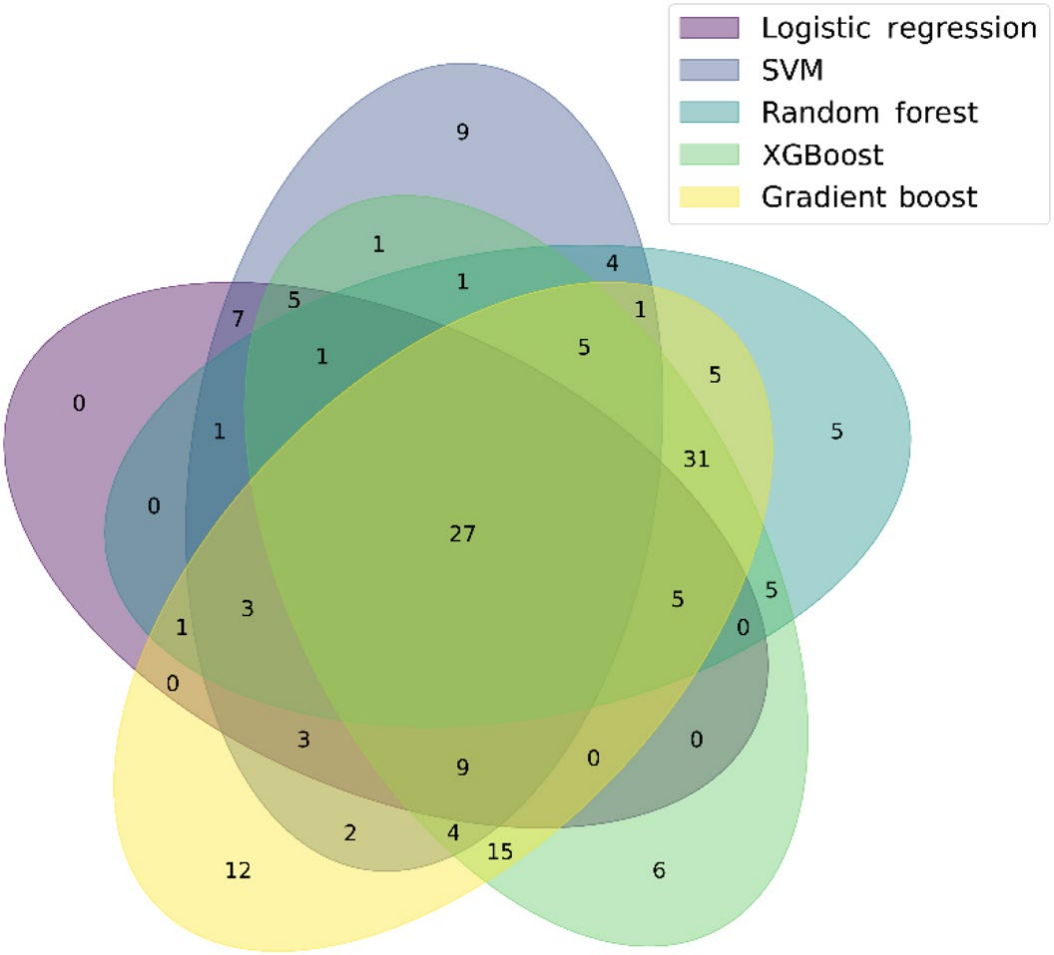
Comparison of features shared among 5 machine learning models. *SVM* support vector machine; *XGBoost* extreme gradient boosting.

Since different models have different advantages in data classification which affects the feature selection results of RFECV method, we attempted to use a Venn diagram to find shared features identified from 5 models through RFECV (Fig. 6) to understand how feature sharing could affect the adopted models. The decision tree model was excluded because of its poor predictive power in predicting LAA patients in both training and testing dataset. We found that 27 features were shared among the 5 models (LR, SVM, RF, XGBoost, and the gradient boosting). Of these features, 11 were clinical factors and 16 were serum metabolites (Supplementary Table 4).

**Figure 7:**
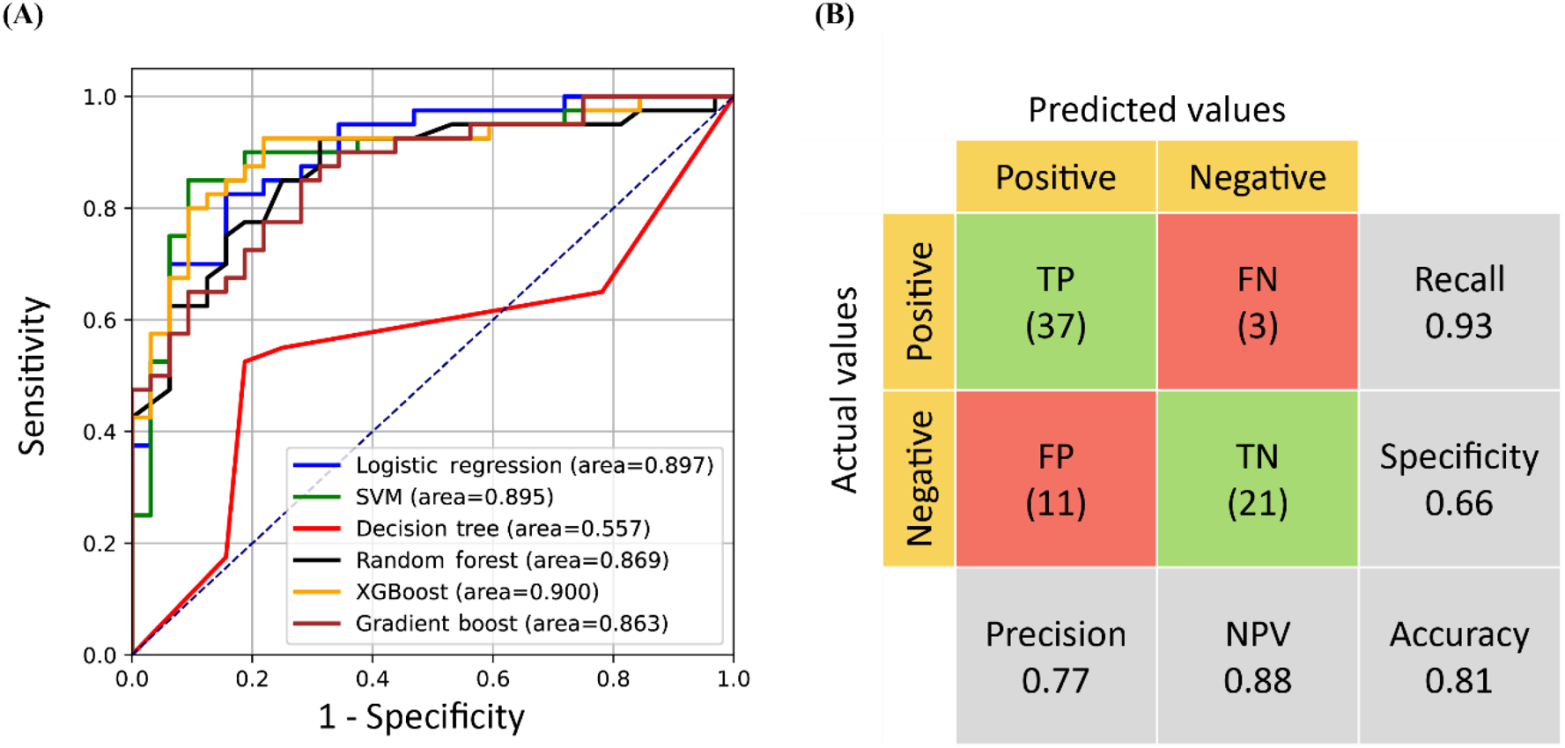
Performance of the 6 predictive models when using the 27 shared features for training: (**A**) receiver operating characteristic curves of the five models for the external validation set and (**B**) confusion matrix for the external validation set when using the LR model. *FN* false negative; *FP* false positive; *NPV* negative predictive value; *SVM* support vector machine; *TN* true negative; *TP* true positive; *XGBoost* extreme gradient boosting.

After training, LR model still exhibited the best AUC (0.93 ± 0.10), followed by SVM model (AUC: 0.91 ± 0.07), RF model (AUC: 0.90 ± 0.13), XGBoost model (AUC: 0.90 ± 0.10) and gradient boost model (AUC: 0.90 ± 0.11) (Table 5). For the external validation set, except for decision tree (AUC: 0.55), the other 5 models exhibited good performance with AUC ≈ 0.9 [Fig. 7A]. The LR model could correctly classified 58 out of 72 patients (accuracy: 0.81), with 3 LAA patients being misclassified as normal controls and 11 normal controls being misclassified as LAA patients [Fig. 7B].

**Table 5:** Performance of five predictive models using 27 shared features.

Model	Accuracy	AUC	Recall	Precision
Logistic regression	0.82 (± 0.16)	0.93 (± 0.10)	0.80 (± 0.22)	0.81 (± 0.17)
SVM	0.83 (± 0.09)	0.91 (± 0.07)	0.85 (± 0.16)	0.81 (± 0.10)
Random forest	0.82 (± 0.22)	0.90 (± 0.13)	0.80 (± 0.26)	0.82 (± 0.24)
XGBoost	0.80 (± 0.15)	0.90 (± 0.10)	0.78 (± 0.31)	0.81 (± 0.16)
Gradient boost	0.81 (± 0.13)	0.90 (± 0.11)	0.80 (± 0.25)	0.80 (± 0.16)
Mean	0.816 (± 0.15)	0.908 (± 0.10)	0.806 (± 0.24)	0.81 (± 0.16)

*AUC* area under the receiver operating characteristic curve; *SVM* support vector machine; *XGBoost* extreme gradient boosting.

### Performance comparison with other research

Several classification algorithms have been developed in various studies for predicting cardiovascular risk using different input data types and methods. Table 6 provides a comparison of their accuracy and AUC values. However, significant progress had not been made until 2020 when Du et al. applied six machine learning methods to electronic health records (EHR) data for predicting cardiovascular risk, achieving an AUC of 0.94, accuracy of 0.87, and Recall of 0.82. In 2022, Huang et al. enhanced a logistic regression model with multivariate methods and clinical data, achieving an AUC of 0.93 for identifying carotid atherosclerosis (CAS). Nevertheless, clinical factors or EHR data typically reflect specific physiological markers and may not fully represent the overall health status of the patient, which could limit their ability to predict certain complex diseases. Additionally, there may be overlapping blood test values among different diseases, which could increase the risk of confusion and misinterpretation when predicting complex conditions. Therefore, in this study, we utilized clinical data combined with metabolomics data to develop a predictive model that could more accurately reflect the actual physical state of the patient.

**Table 6:** Comparison of predictive performance with other studies.

Year	Input data	Method	AUC	Accuracy	Recall (Sensitivity)	References
Ours	Clinical data + Metabolomics data (27 features)	5 machine learning models (LR, SVM, RF, XGBoost, Gradient Boost)	0.93	0.81	0.93	–
2017	Clinical data	4 machine learning models (RF, LR, Gradient Boosting, Neural Networks)	0.76	–	0.67	^69^
2019	Clinical data	ANN and KNN	–	0.96	0.97	^70^
2020	Clinical data + Serum biomarkers	Multivariate logistic regression model	0.83	–	–	^71^
2020	Coronary angiography	Gradient Boosted Trees (GBT) algorithm	0.76	0.73	0.67	^72^
2020	EHRs	6 machine learning models (LR, DT, KNN, SVM, RF, XGBoost)	0.94	0.87	0.82	^73^
2021	Clinical data	Support vector machine	–	0.91	0.80	^74^
2022	Clinical data	Multivariate logistic regression analysis	0.93	–	–	^75^
2022	Clinical data	Random Forest Classifier	0.84	–	–	^76^
2022	Clinical data	8 ML models (LR, DT, RF, XGBoost, LightGBM, GBDT, SVM, Neural Networks)	0.85	0.80	0.82 (RF)	^76^

*AUC* area under the receiver operating characteristic curve.

## Discussion

In this study, we considered common vascular risk factors and serum metabolites for predicting extracranial LAA. The cross-comparison results of 18 models (3 scales of input features in 6 commonly used machine learning algorithms) showed the combination of clinical factors and metabolites had better predictive performance across different training algorithms, and the robustness could be observed through using the external validation set [Fig. 3C] which indicated LR was the best fit algorithm for LAA prediction (AUC: 0.89).

Previous study in Kazakh Chinese suggested that using clinical factors with SVM (AUC: 0.86) and LR (AUC: 0.87) algorithms had better performance in predicting cardiovascular disease^48^. Another study in Northern China found that training the models using clinical factors alone could limit the model performance with the AUC range between 0.55 and 0.67 in LR, RF and SVM models in predicting acute ischemic stroke^49^. However, many studies have pointed out that tree-based algorithms such as RF and gradient boosting were much more useful than regression-based algorithms in predicting large vessel occlusion with advanced measurements and pretreatment scoring methods^50–52^. For example, Alanazi et al. had trained a RF model with an AUC of 0.97 in Caucasian^53^; Nishi and his colleagues had trained a RF model with an AUC of 0.85 in Japanese^54^ ; and Wang et al. had trained a gradient boosting model with an AUC of 0.83 in Han Chinese^55^. Our study demonstrated the performance metrics could be improved if a feature selection step was added and both clinical factors and metabolites could be combined for disease prediction. Also, the performance could be improved if the feature number was reduced from 193 to 62 in the LR model with the mean AUC arranged from 0.89 ± 0.12 [Table 3 and Fig. 3C] to 0.96 ± 0.03 [Fig. 5A].

The 62 features selected through RFECV comprised 15 clinical factors and 47 metabolites (Supplementary Table 3). Among these clinical factors, some may be related to atherosclerosis. For instance, age usually reflects lifestyle and physical condition^56^. Studies have found that older individuals exhibit higher collagen deposition and vascular stiffness^50^, which might contribute to subsequent hypertension and the expression of several proinflammatory molecules that ultimately promote atherosclerosis^51^. High levels of blood pressure, cholesterol, and glucose may aggravate the risk of atherosclerosis by increasing the rate of plaque buildup and causing endothelial injury or dysfunction^52,57^. The balance among serum lipids, platelets, hemodynamic factors, and the blood vessel wall may influence the development of atherosclerosis^58^.

As for the 47 metabolites, the association with atherosclerosis was less discussed. We then conducted a pathway analysis on the 47 metabolites by using MetaboAnalyst v5.0 (Supplementary Table 5)^59^. The pathway enrichment analysis indicated that these metabolites were associated with aminoacyl-tRNA biosynthesis and lipid metabolism (*p* < 0.05). We found that the levels of half of the examined PCs were significantly lower in the patients with LAA than normal controls. The reasons for this result remain unclear. Studies have examined the prognostic values of PCs and lysoPCs and have observed a lower level in patients with coronary and peripheral arterial disease^60,61^. Moxon et al. suggested that a lower level of serum PC might lead to an increased rate of low-density lipoprotein oxidation, increased foam cell formation, and reduced cholesterol efflux^62^. Moreover, amino acid metabolism has strong relationships with several types of cardiovascular disease^63^. For example, valine, leucine, and isoleucine can ameliorate cell metabolic processes through mitochondrial biogenesis, influence macrophage foam cells, and alter lipid metabolism^64^. Tryptophan levels are identified to be lower in patients with atheromatous plaque or coronary heart disease than healthy individuals^65^. Many metabolites of tryptophan and arginine play a role in regulating athero-inflammation and atherogenesis^66^. Evidence suggests that disturbances in the balance and transmission of immune signaling might also affect LAA^67^.

This study is the first to integrate multi-model testing and the RFECV method in training and testing to create LAA prediction model. We eliminated irrelevant features, improved the learning accuracy of the models, and determined the best fitting model for our dataset. We found that sharing relevant features between models provided additional class-specific information that helped reduce the computational complexity and improve the recall rate efficiently. We used the RFECV method to identify specific features for LAA prediction. Even though we had trained a good LR model to predict LAA based on 62 features. To simplify the prediction model, we removed the decision tree and selected the 27 features shared among LR, SVM, RF, XGBoost, and gradient boosting. The performance indicated that using these 27 features could improve the 4 performance metrics to > 0.80 compared to the models using 193 features. However, if focused on the LR model, using the 27 features had a higher recall but lower precision compared to using the 62 features identified through RFECV. Our study may suggest using the 27 features in LR model could be a suitable test for screening LAA^67^.

LAA carries a high risk of cerebrovascular disease and results in worse outcome than other ischemic stroke subtypes^1^. The benefit of our study is that we developed a simple algorithm to screen LAA by using clinical factors and a simple blood test for metabolite profile instead of using a complicated neuroimaging study. Our study may help to early predict and prevent the progression of LAA. There is also potential that the use of machine learning may help to set up algorithm to solve clinical issues to improve the healthcare system. The predictive models created by machine learning can be applied in other medical conditions to improve disease diagnosis and management.

However, this study had several limitations. First, this was a single-center-based retrospective study, and external validation is required to determine the optimal model’s stability and population drift. Second, statistical problems might occur when machine learning is used on small data sets^68^. Our data set was balanced between the patient and control groups, and the results of tenfold cross-validation on an external validation set indicated that our model provided nearly unbiased performance estimates. Nevertheless, further large-scale cohort estimation is required before the clinical application of the model. Third, the metabolites were analyzed using a commercial kit (the Absolute IDQ®p180 kit; Biocrates Life Science, AG, Innsbruck, Austria). Different quantitative methods have different resolutions; thus, the universal usage of the optimal model might not be possible.

In conclusion, our study indicated that a high-accuracy LAA prediction model can be developed by integrating multi-model testing and the RFECV method. Compared to clinical factors or metabolites alone, data on a combination of appropriate clinical factors and metabolites provide a real-time understanding of an individual’s LAA status. The machine learning process used in this study can be adopted by researchers in future studies to construct high-performance prediction models for other complex diseases.

For future application, the LR model demonstrates accurate prediction of LAA thrombosis when compared to other scoring methods. This model can effectively assess risks in patients with LAA. Additionally, our study recognizes the significance of specific metabolite biomarkers in predicting atherosclerosis. It is our hope that these findings and recommendations will greatly contribute to optimizing treatment strategies for patients with LAA and hold immense significance in the prevention of LAA.

### Supplementary Information

Supplementary Information.

## Data Availability

The datasets generated and/or analyzed during the current study are available in the Google drive repository: https://drive.google.com/file/d/1KL-8Vtz4AFk342WZq_TReGReBVInxXAv/view?usp=share_link.
