# Supplementary material for: Machine learning approaches for biomarker discovery to predict large-artery atherosclerosis
Source: Sci Rep. 2023 Sep 13;13:15139. doi: 10.1038/s41598-023-42338-0 (PMC10499778; doi:10.1038/s41598-023-42338-0)
Supplement: Supplementary file 1 — Supplementary Information. [file 41598_2023_42338_MOESM1_ESM.docx]

**Machine Learning Approaches for Biomarker discovery to Predict Large-Artery Atherosclerosis**

**Ting-Hsuan Sun^1†^, Chia-Chun Wang^1†^, Ya-Lun Wu^1^, Kai-Cheng Hsu^1, 2, 3*^, Tsong-Hai Lee^4*^**

^1^ Artificial Intelligence Center, China Medical University Hospital, Taichung, Taiwan

^2^ Department of Neurology, China Medical University Hospital, Taichung, Taiwan

^3^ Department of Medicine, China Medical University, Taichung, Taiwan

^4^ Stroke Center and Department of Neurology, Linkou Chang Gung Memorial Hospital, and College of Medicine, Chang Gung University, Taoyuan, Taiwan

^†^ These authors contributed equally to this work.

***** Corresponding authors**:** Kai-Cheng Hsu (e-mail: D35842@mail.cmuh.org.tw) and Tsong-Hai Lee (e-mail: thlee@adm.cgmh.org.tw) contributed equally to the manuscript.

**Supplementary methods**

**A. Data preprocessing:**

1. Missing data handling: Mean imputation

(1). R package:

*simple.impute.data.frame()*

(2). Package resource:

<https://www.rdocumentation.org/packages/useful/versions/1.2.6/topics/simple.impute.data.frame>

(3). Codes in our project:

simple.impute.data.frame(LAA.0328.clinical.miRNA,mean)

1. Label encoding + Grouping:

(1). Python package:

*numpy.where(condition, [x, y, ]/)*

(2). Package resource:

<https://numpy.org/doc/stable/reference/generated/numpy.where.html>

(3). Codes in our project:

laa_clean['Group'] = np.where(laa_clean['Group']== "LAA", 1, 0)

1. Training Testing data split:

(1). Python package:

*train_test_split()*

(2). Package resource:

<https://scikit-learn.org/stable/modules/generated/sklearn.model_selection.train_test_split.html>

(3). Codes in our project:

X_train, X_test, y_train, y_test = train_test_split(x, y, test_size = 0.2, random_state =2018, shuffle = True)

1. Stratified *k*-fold:

(1). Python package:

*sklearn.model_selection.cross_validate()*

(2). Package resource:

<https://scikit-learn.org/stable/modules/generated/sklearn.model_selection.cross_validate.html>

(3). Codes in our project:

scoring=['accuracy','roc_auc','recall','precision','f1']

cross_validate(classifier,X,y,cv=10,scoring=scoring)

1. RFECV feature selection:

(1). Python package:

*RFECV ()*

(2). Package resource:

<https://scikit-learn.org/stable/modules/generated/sklearn.feature_selection.RFECV.html>

(3). Codes in our project:

RFECV(LogisticRegression(max_iter=3000),cv=cv_rfe,scoring="roc_auc")

1. Machine learning models:

(1). Python package:

Scikit-Learn Machine Learning in Python

1. Logistic Regression:

*class sklearn.linear_model.LogisticRegression()*

1. Support Vector Machine:

*class sklearn.svm.SVC()*

1. Decision Tree:

*tree.DecisionTreeClassifier()*

1. Random Forest:

*class sklearn.ensemble.RandomForestClassifier()*

1. XGBoost:

*xgb.XGBRegressor()*

1. Gradient Boost

*class sklearn.ensemble.GradientBoostingClassifier()*

(2). Package resource:

Scikit-learn:

<https://scikit-learn.org/stable/index.html>

1. Logistic Regression:

<https://scikit-learn.org/stable/modules/generated/sklearn.linear_model.LogisticRegression.html>

1. SVM:

<https://scikit-learn.org/stable/modules/generated/sklearn.svm.SVC.html>

1. Decision Tree:

<https://scikit-learn.org/stable/modules/tree.html>

1. Random Forest:

<https://scikit-learn.org/stable/modules/generated/sklearn.ensemble.RandomForestRegressor.html>

1. XGBoost:

<https://xgboost.readthedocs.io/en/stable/python/python_api.html>

1. Gradient Boost:

<https://scikit-learn.org/stable/modules/generated/sklearn.ensemble.GradientBoostingClassifier.html>

(3). Codes in our project:

A. classifier_LR = LogisticRegression(max_iter=3000)

B. SVC(kernel='rbf',probability=True , class_weight = 'balanced')

C. DecisionTreeClassifier(max_depth=6)

D. RandomForestClassifier()

E. XGBClassifier(objective='binary:logistic',

                          booster='gbtree',

                          eval_metric='auc',

                          tree_method='hist',

                          grow_policy='lossguide',

                          use_label_encoder=None)

F. GradientBoostingClassifier()

1. Model evaluation:

(1). Python package:

Accuracy: *sklearn.metrics.accuracy_score()*

AUC: *sklearn.metrics.auc()*

Recall: *sklearn.metrics.recall_score()*

Precision: *sklearn.metrics.precision_score()*

(2). Package resource:

Accuracy:

<https://scikit-learn.org/stable/modules/generated/sklearn.metrics.accuracy_score.html>

AUC:

<https://scikit-learn.org/stable/modules/generated/sklearn.metrics.auc.html>

Recall:

<https://scikit-learn.org/stable/modules/generated/sklearn.metrics.recall_score.html>

Precision:

<https://scikit-learn.org/stable/modules/generated/sklearn.metrics.precision_score.html>

(3). Codes in our project:

1. accuracy = metrics.accuracy_score(y_test, predicted)
2. auc_test = metrics.roc_auc_score(y_test, predicted_prob_LR)
3. recall = metrics.recall_score(y_test, predicted)
4. precision = metrics.precision_score(y_test, predicted)

**B. Machine Learning Models:**

**LR** is a supervised learning technique used to address classification issues and determine the likelihood of a binary (yes/no) occurrence. Whatever the variable is dichotomous or categorical, the logistic function use a S-shaped curve to transform data into a value between 0 and 1 for classification issues [1].

**SVM** was first proposed by Corinna et al. [2]. Processing nonlinear, small-sample, and high-dimensional pattern recognition problems with SVM provides various benefits. It offers a great generalization ability for unknown samples because the partitioning hyperplane may ensure that the extreme solution is a global optimal solution rather than a local minimal value and has a solid theoretical foundation [3].

**Decision tree algorithm** is a common type of machine learning algorithm in which decisions are made according to a tree structure. A decision tree typically has a root node, a number of internal nodes, and a number of leaf nodes. The root node includes all of the samples, and each node's samples are separated into subnodes based on the outcomes of an attribute test. The sequence of decision tests corresponds to the route from the root node to the last leaf node [3, 4].

**RF** is an extension of the bagging method [5], which is a typical ensemble learning method. Bagging often entails processing chores using a straightforward voting system. A decision tree algorithm serves as the foundation learner for RF, and during decision tree training, random attribute selection is included. For a variety of real-world data, RF offers reliable performance, and is easily understood [3]. It has shown good performance in applications like disease prediction, gene selection, and picture recognition [6-8].

**XGBoost** is a novel gradient boosting ensemble learning method. In this method, machine learning is implemented under the gradient boosting framework with high efficiency, flexibility, and portability[9]. Tree boosting is an efficient and widely used machine learning method that is a type of boosted ensemble learning [10]. The second-order Taylor expansion of the loss function is used by the XGBoost model, and a regularization function is added to this expansion to strike a compromise between the model's complexity and loss function reduction. This approach attempts to avoid overfitting to some extent by looking for the overall ideal solution [3]. The gradient tree boosting algorithm used by XGBoost is to increase its speed and accuracy.

**Gradient boosting** is a fast and accurate machine-learning-based prediction method that is particularly well suited for large and complicated datasets. Gradient boosting redefines boosting as a numerical optimization problem with the objective of minimizing the loss function by incorporating a weak learner via gradient descent. In order to reduce the overall error of the strong learner, the contribution of each weak learner to the final prediction is based on a gradient optimization procedure. Gradient boosting focuses on existing underperforming learners [11].

**C. Statistical Analysis:**

In the models of this study, we set the maximum number of iterations as 3,000 and added a penalty term (L2) to the loss function in our LR model. For our SVM model, the radial basis function was used as the kernel function, the regularization parameter (C) was 1.0, and the class weight was set as “balanced.” For the decision tree model, the maximum depth of a tree was 6. For the RF and gradient boosting algorithms, default parameter settings were used. For the XGBoost algorithm, we used the tree construction algorithm (tree_method) as “hist”, and nodes with the highest loss change were added to the tree (grow_policy).

The input data was split into a training set and an external validation set, following an 8:2 ratio. Subsequently, the training set was divided into k equal parts for k-fold internal cross-validation. During each of the k iterations, one part of the training set was designated for internal validation, while the remaining k – 1 parts were employed for training the models. This approach facilitated thorough evaluation and model training using the training sets. Lastly, the performance of the models was evaluated using the external validation set. This procedure was repeated until each of the k subsets had been served as the validation set. The average of the k performance measurements was the cross-validated performance [12]. In this study, we conducted internal stratified 10-fold cross-validation in the training set to estimate the performance of the models [13]. And the final performance of the models was evaluated using the external validation set. The RFECV algorithm was used to determine the contributions of features to the predictions classified into the LAA and “control” categories.

**Supplementary result**


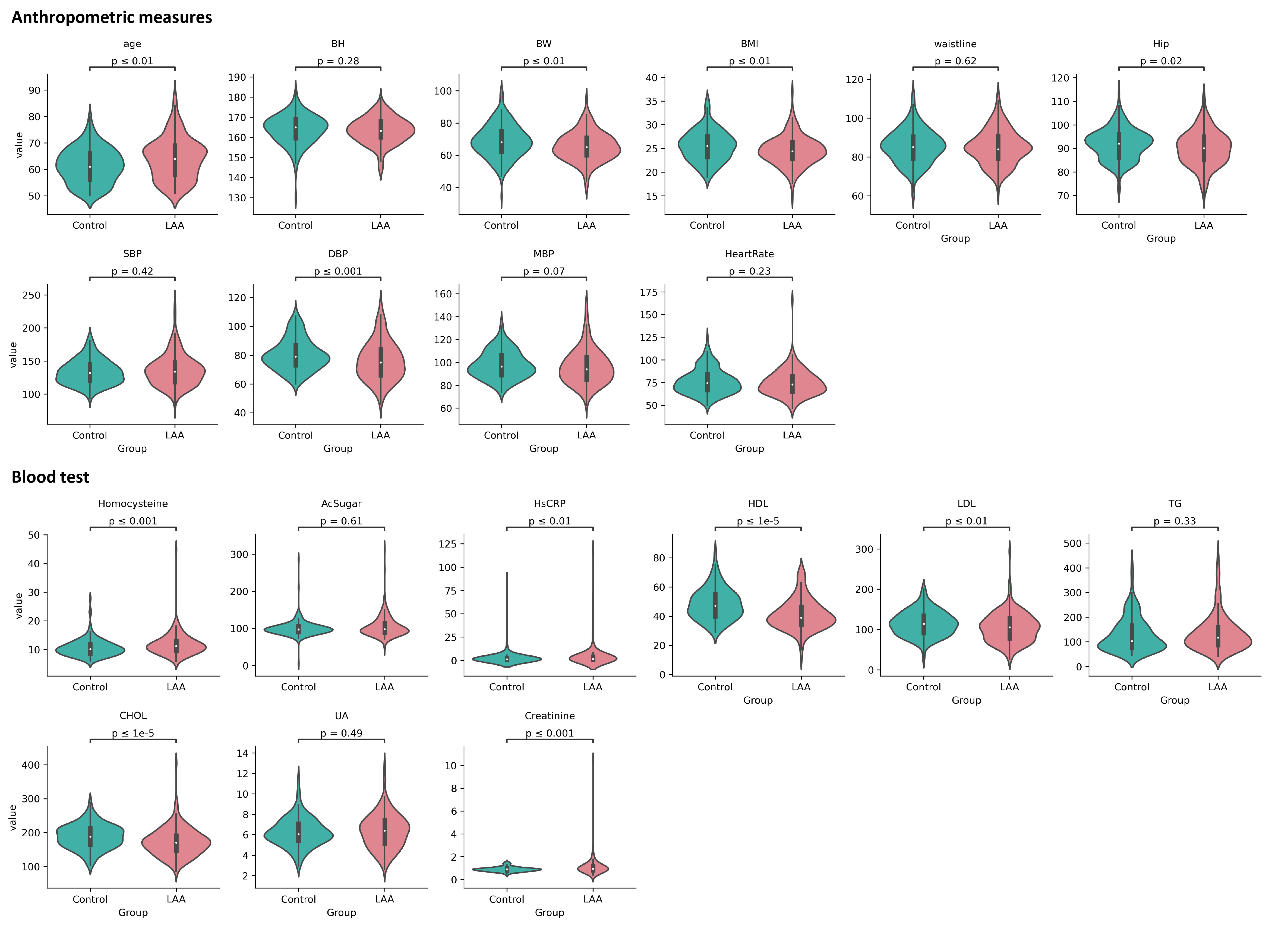


**Supplementary Fig. S1.** Violin plots showing the distributions of clinical factors for the control and LAA groups. LAA: large-artery atherosclerosis.


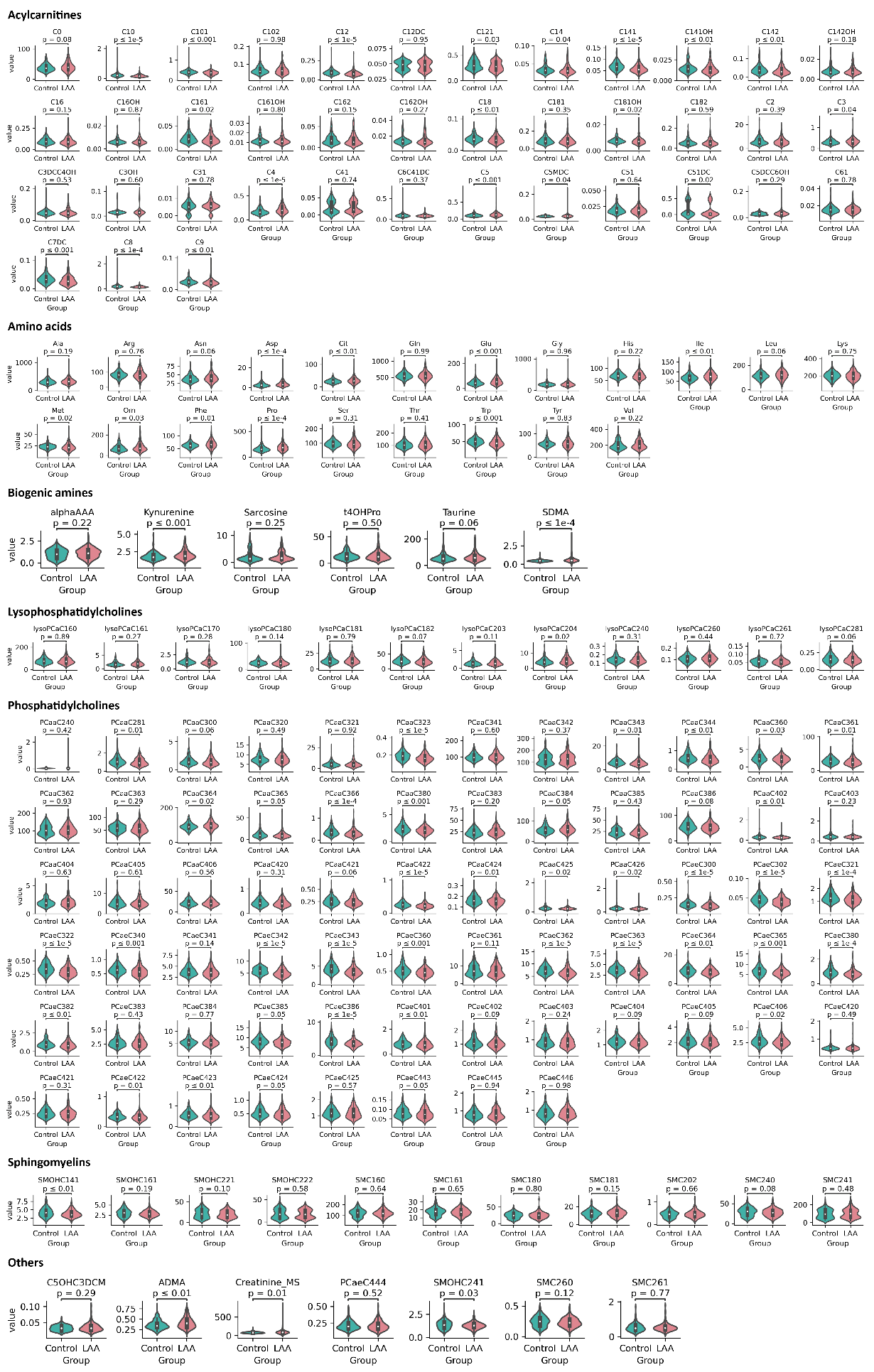


**Supplementary Fig. S2.** Violin plots showing the distributions of serum metabolites for the control and LAA groups. LAA: large-artery atherosclerosis.

**Supplementary table 1**. Serum metabolites in the 359 study participants. LAA: large-artery atherosclerosis

| **Class** | **Variable** | **N** | **LAA,** N = 176*^1^* | **Control**, N = 183*^1^* | ***p*-value***^2^* |
| --- | --- | --- | --- | --- | --- |
| Acylcarnitines | C0 | 359 | 40 (31, 49) | 36 (30, 45) | 0.078 |
|  | C10 | 359 | 0.14 (0.11, 0.22) | 0.20 (0.15, 0.26) | < 0.001 |
|  | C101 | 305 | 0.36 (0.30, 0.42) | 0.40 (0.35, 0.46) | < 0.001 |
|  | C102 | 359 | 0.063 (0.047, 0.079) | 0.060 (0.052, 0.076) | > 0.9 |
|  | C12 | 359 | 0.074 (0.060, 0.097) | 0.089 (0.072, 0.110) | < 0.001 |
|  | C12DC | 305 | 0.048 (0.039, 0.054) | 0.049 (0.040, 0.054) | > 0.9 |
|  | C121 | 359 | 0.28 (0.20, 0.38) | 0.30 (0.23, 0.38) | 0.032 |
|  | C14 | 305 | 0.030 (0.026, 0.038) | 0.032 (0.027, 0.038) | 0.045 |
|  | C141 | 359 | 0.055 (0.047, 0.069) | 0.068 (0.056, 0.080) | < 0.001 |
|  | C141OH | 305 | 0.012 (0.010, 0.016) | 0.014 (0.011, 0.017) | 0.005 |
|  | C142 | 359 | 0.028 (0.018, 0.047) | 0.035 (0.024, 0.051) | 0.003 |
|  | C142OH | 305 | 0.007 (0.006, 0.010) | 0.007 (0.006, 0.010) | 0.2 |
|  | C16 | 359 | 0.09 (0.07, 0.12) | 0.09 (0.07, 0.12) | 0.2 |
|  | C16OH | 305 | 0.0060 (0.0050, 0.0073) | 0.0060 (0.0050, 0.0070) | 0.9 |
|  | C161 | 359 | 0.019 (0.014, 0.026) | 0.022 (0.016, 0.029) | 0.018 |
|  | C161OH | 305 | 0.011 (0.010, 0.013) | 0.011 (0.010, 0.013) | 0.8 |
|  | C162 | 305 | 0.013 (0.009, 0.023) | 0.016 (0.010, 0.025) | 0.15 |
|  | C162OH | 305 | 0.012 (0.011, 0.016) | 0.013 (0.011, 0.015) | 0.3 |
|  | C18 | 305 | 0.032 (0.026, 0.042) | 0.036 (0.030, 0.043) | 0.004 |
|  | C181 | 359 | 0.08 (0.06, 0.11) | 0.08 (0.06, 0.11) | 0.3 |
|  | C181OH | 305 | 0.0070 (0.0060, 0.0083) | 0.0070 (0.0070, 0.0090) | 0.019 |
|  | C182 | 359 | 0.051 (0.037, 0.070) | 0.052 (0.038, 0.067) | 0.6 |
|  | C2 | 359 | 5.60 (3.71, 7.34) | 5.34 (4.19, 7.80) | 0.4 |
|  | C3 | 359 | 0.33 (0.24, 0.42) | 0.29 (0.23, 0.38) | 0.039 |
|  | C3DCC4OH | 359 | 0.046 (0.038, 0.056) | 0.046 (0.039, 0.058) | 0.5 |
|  | C3OH | 305 | 0.014 (0.012, 0.017) | 0.014 (0.012, 0.017) | 0.6 |
|  | C31 | 305 | 0.006 (0.005, 0.007) | 0.005 (0.004, 0.007) | 0.8 |
|  | C4 | 359 | 0.20 (0.16, 0.28) | 0.16 (0.13, 0.20) | < 0.001 |
|  | C41 | 305 | 0.015 (0.009, 0.036) | 0.013 (0.008, 0.039) | 0.7 |
|  | C6C41DC | 359 | 0.08 (0.07, 0.10) | 0.09 (0.07, 0.11) | 0.4 |
|  | C5 | 359 | 0.12 (0.09, 0.16) | 0.10 (0.08, 0.13) | < 0.001 |
|  | C5MDC | 305 | 0.025 (0.022, 0.030) | 0.024 (0.020, 0.029) | 0.038 |
|  | C51 | 305 | 0.019 (0.015, 0.022) | 0.019 (0.016, 0.022) | 0.6 |
|  | C51DC | 305 | 0.01 (0.01, 0.02) | 0.02 (0.01, 0.43) | 0.019 |
|  | C5DCC6OH | 305 | 0.029 (0.020, 0.037) | 0.029 (0.019, 0.036) | 0.3 |
|  | C61 | 305 | 0.015 (0.012, 0.018) | 0.015 (0.013, 0.018) | 0.8 |
|  | C7DC | 359 | 0.026 (0.017, 0.039) | 0.032 (0.025, 0.044) | < 0.001 |
|  | C8 | 359 | 0.16 (0.13, 0.21) | 0.20 (0.15, 0.25) | < 0.001 |
|  | C9 | 305 | 0.020 (0.016, 0.026) | 0.023 (0.019, 0.028) | 0.002 |
| Amino acids | Ala | 359 | 321 (254, 390) | 299 (248, 367) | 0.2 |
|  | Arg | 359 | 79 (65, 102) | 82 (69, 95) | 0.8 |
|  | Asn | 359 | 39 (33, 48) | 37 (32, 45) | 0.057 |
|  | Asp | 359 | 3.03 (2.08, 5.20) | 2.30 (1.40, 3.70) | < 0.001 |
|  | Cit | 359 | 26 (20, 35) | 24 (18, 29) | 0.003 |
|  | Gln | 359 | 544 (478, 627) | 542 (482, 617) | > 0.9 |
|  | Glu | 359 | 53 (38, 74) | 43 (33, 60) | < 0.001 |
|  | Gly | 359 | 182 (149, 226) | 185 (154, 214) | > 0.9 |
|  | His | 359 | 68 (59, 79) | 69 (63, 78) | 0.2 |
|  | Ile | 359 | 76 (61, 89) | 69 (58, 79) | 0.002 |
|  | Leu | 359 | 122 (95, 144) | 112 (95, 134) | 0.061 |
|  | Lys | 359 | 198 (167, 234) | 202 (172, 234) | 0.8 |
|  | Met | 359 | 21 (18, 27) | 23 (20, 27) | 0.018 |
|  | Orn | 359 | 68 (54, 102) | 65 (47, 88) | 0.028 |
|  | Phe | 359 | 66 (57, 77) | 62 (57, 69) | 0.011 |
|  | Pro | 359 | 184 (141, 231) | 150 (126, 188) | < 0.001 |
|  | Ser | 359 | 93 (75, 113) | 96 (81, 111) | 0.3 |
|  | Thr | 359 | 104 (83, 130) | 102 (83, 119) | 0.4 |
|  | Trp | 359 | 45 (38, 54) | 50 (44, 57) | < 0.001 |
|  | Tyr | 359 | 58 (46, 68) | 57 (50, 64) | 0.8 |
|  | Val | 359 | 193 (168, 248) | 184 (164, 233) | 0.2 |
| Biogenic amines | alphaAAA | 359 | 1.11 (0.66, 1.48) | 1.00 (0.54, 1.44) | 0.2 |
|  | Kynurenine | 359 | 1.90 (1.60, 2.32) | 1.70 (1.46, 2.08) | < 0.001 |
|  | Sarcosine | 359 | 1.54 (1.08, 3.40) | 1.36 (1.00, 3.01) | 0.2 |
|  | t4OHPro | 359 | 12 (8, 18) | 13 (9, 18) | 0.5 |
|  | Taurine | 359 | 58 (44, 75) | 51 (41, 69) | 0.061 |
|  | SDMA | 359 | 0.50 (0.42, 0.62) | 0.47 (0.40, 0.51) | < 0.001 |
| Lysophosphatidylcholines | lysoPCaC160 | 359 | 72 (58, 98) | 75 (58, 92) | 0.9 |
|  | lysoPCaC161 | 359 | 1.79 (1.26, 2.30) | 1.51 (1.29, 2.18) | 0.3 |
|  | lysoPCaC170 | 359 | 1.15 (0.88, 1.50) | 1.16 (0.94, 1.50) | 0.3 |
|  | lysoPCaC180 | 359 | 23 (18, 29) | 24 (20, 30) | 0.14 |
|  | lysoPCaC181 | 359 | 12.4 (9.2, 16.6) | 12.1 (9.5, 16.0) | 0.8 |
|  | lysoPCaC182 | 359 | 23 (17, 32) | 25 (19, 33) | 0.071 |
|  | lysoPCaC203 | 359 | 1.36 (1.04, 2.00) | 1.33 (0.95, 1.75) | 0.11 |
|  | lysoPCaC204 | 359 | 4.55 (3.14, 6.37) | 3.94 (3.01, 5.27) | 0.018 |
|  | lysoPCaC240 | 359 | 0.13 (0.11, 0.17) | 0.14 (0.12, 0.16) | 0.3 |
|  | lysoPCaC260 | 359 | 0.109 (0.089, 0.135) | 0.108 (0.088, 0.127) | 0.4 |
|  | lysoPCaC261 | 359 | 0.052 (0.041, 0.064) | 0.054 (0.040, 0.066) | 0.7 |
|  | lysoPCaC281 | 359 | 0.14 (0.11, 0.18) | 0.15 (0.11, 0.20) | 0.062 |
| Phosphatidylcholines | PCaaC240 | 359 | 0.032 (0.026, 0.044) | 0.031 (0.026, 0.040) | 0.4 |
|  | PCaaC281 | 359 | 0.92 (0.69, 1.32) | 1.02 (0.82, 1.33) | 0.012 |
|  | PCaaC300 | 359 | 1.25 (0.89, 1.81) | 1.35 (1.04, 1.89) | 0.064 |
|  | PCaaC320 | 359 | 7.43 (5.99, 8.93) | 7.39 (6.44, 8.76) | 0.5 |
|  | PCaaC321 | 359 | 4.2 (2.9, 6.2) | 4.3 (2.8, 6.3) | > 0.9 |
|  | PCaaC323 | 359 | 0.16 (0.14, 0.19) | 0.19 (0.16, 0.22) | < 0.001 |
|  | PCaaC341 | 359 | 99 (86, 114) | 97 (87, 114) | 0.6 |
|  | PCaaC342 | 359 | 130 (91, 159) | 126 (90, 157) | 0.4 |
|  | PCaaC343 | 359 | 5.40 (4.27, 7.00) | 6.05 (4.70, 7.74) | 0.014 |
|  | PCaaC344 | 359 | 0.51 (0.38, 0.69) | 0.60 (0.46, 0.75) | 0.001 |
|  | PCaaC360 | 359 | 2.22 (1.55, 2.91) | 2.35 (1.72, 3.37) | 0.032 |
|  | PCaaC361 | 359 | 24 (18, 32) | 27 (21, 35) | 0.013 |
|  | PCaaC362 | 359 | 104 (86, 124) | 102 (81, 128) | > 0.9 |
|  | PCaaC363 | 359 | 57 (45, 71) | 60 (49, 70) | 0.3 |
|  | PCaaC364 | 359 | 96 (85, 110) | 91 (79, 103) | 0.024 |
|  | PCaaC365 | 359 | 8 (6, 13) | 10 (7, 15) | 0.046 |
|  | PCaaC366 | 359 | 0.28 (0.19, 0.37) | 0.34 (0.24, 0.47) | < 0.001 |
|  | PCaaC380 | 359 | 2.11 (1.70, 2.56) | 2.32 (1.90, 2.91) | < 0.001 |
|  | PCaaC383 | 359 | 24 (19, 32) | 23 (18, 30) | 0.2 |
|  | PCaaC384 | 359 | 58 (46, 74) | 54 (43, 67) | 0.05 |
|  | PCaaC385 | 359 | 22 (18, 28) | 22 (18, 31) | 0.4 |
|  | PCaaC386 | 359 | 54 (43, 65) | 56 (46, 70) | 0.076 |
|  | PCaaC402 | 359 | 0.26 (0.20, 0.32) | 0.28 (0.22, 0.36) | 0.002 |
|  | PCaaC403 | 359 | 0.42 (0.34, 0.49) | 0.43 (0.34, 0.52) | 0.2 |
|  | PCaaC404 | 359 | 1.98 (1.46, 2.46) | 1.89 (1.48, 2.33) | 0.6 |
|  | PCaaC405 | 359 | 4.88 (3.84, 6.07) | 4.77 (3.89, 6.39) | 0.6 |
|  | PCaaC406 | 359 | 20 (16, 26) | 20 (17, 27) | 0.6 |
|  | PCaaC420 | 359 | 0.40 (0.32, 0.52) | 0.42 (0.34, 0.54) | 0.3 |
|  | PCaaC421 | 359 | 0.23 (0.18, 0.29) | 0.25 (0.20, 0.31) | 0.06 |
|  | PCaaC422 | 359 | 0.21 (0.17, 0.26) | 0.25 (0.21, 0.31) | < 0.001 |
|  | PCaaC424 | 359 | 0.16 (0.13, 0.18) | 0.16 (0.14, 0.19) | 0.015 |
|  | PCaaC425 | 359 | 0.21 (0.17, 0.26) | 0.22 (0.18, 0.30) | 0.023 |
|  | PCaaC426 | 359 | 0.26 (0.21, 0.32) | 0.28 (0.23, 0.36) | 0.02 |
|  | PCaeC300 | 359 | 0.12 (0.10, 0.16) | 0.15 (0.12, 0.17) | < 0.001 |
|  | PCaeC302 | 359 | 0.040 (0.033, 0.047) | 0.046 (0.039, 0.054) | < 0.001 |
|  | PCaeC321 | 359 | 1.11 (0.87, 1.38) | 1.24 (1.04, 1.54) | < 0.001 |
|  | PCaeC322 | 359 | 0.29 (0.23, 0.36) | 0.35 (0.28, 0.43) | < 0.001 |
|  | PCaeC340 | 359 | 0.56 (0.44, 0.67) | 0.63 (0.53, 0.74) | < 0.001 |
|  | PCaeC341 | 359 | 3.56 (2.90, 4.32) | 3.64 (3.12, 4.36) | 0.14 |
|  | PCaeC342 | 359 | 4.81 (3.86, 6.18) | 5.97 (4.89, 7.20) | < 0.001 |
|  | PCaeC343 | 359 | 3.34 (2.73, 4.22) | 4.28 (3.42, 5.22) | < 0.001 |
|  | PCaeC360 | 359 | 0.46 (0.38, 0.55) | 0.51 (0.42, 0.60) | < 0.001 |
|  | PCaeC361 | 359 | 6.4 (4.5, 9.2) | 7.0 (5.0, 9.8) | 0.11 |
|  | PCaeC362 | 359 | 6.25 (5.28, 7.51) | 7.11 (6.16, 8.55) | < 0.001 |
|  | PCaeC363 | 359 | 3.09 (2.56, 3.82) | 3.67 (3.08, 4.34) | < 0.001 |
|  | PCaeC364 | 359 | 7.97 (6.41, 9.73) | 8.88 (7.15, 10.85) | 0.005 |
|  | PCaeC365 | 359 | 5.84 (4.81, 7.02) | 6.71 (5.16, 7.93) | < 0.001 |
|  | PCaeC380 | 359 | 0.78 (0.61, 0.95) | 0.92 (0.71, 1.13) | < 0.001 |
|  | PCaeC382 | 359 | 0.78 (0.54, 1.13) | 0.91 (0.65, 1.22) | 0.009 |
|  | PCaeC383 | 359 | 2.62 (2.12, 3.27) | 2.66 (2.20, 3.19) | 0.4 |
|  | PCaeC384 | 359 | 5.37 (4.56, 6.33) | 5.49 (4.61, 6.31) | 0.8 |
|  | PCaeC385 | 359 | 7.68 (6.18, 8.80) | 7.91 (6.93, 9.41) | 0.045 |
|  | PCaeC386 | 359 | 3.46 (2.91, 4.18) | 4.08 (3.24, 5.04) | < 0.001 |
|  | PCaeC401 | 359 | 0.70 (0.51, 0.94) | 0.78 (0.60, 0.97) | 0.007 |
|  | PCaeC402 | 359 | 0.96 (0.82, 1.21) | 1.04 (0.87, 1.29) | 0.09 |
|  | PCaeC403 | 359 | 0.82 (0.65, 1.04) | 0.83 (0.67, 1.06) | 0.2 |
|  | PCaeC404 | 359 | 1.16 (0.97, 1.37) | 1.21 (1.04, 1.41) | 0.086 |
|  | PCaeC405 | 359 | 2.01 (1.63, 2.51) | 2.07 (1.74, 2.59) | 0.086 |
|  | PCaeC406 | 359 | 2.54 (2.13, 3.05) | 2.68 (2.26, 3.26) | 0.025 |
|  | PCaeC420 | 359 | 0.48 (0.41, 0.55) | 0.48 (0.42, 0.54) | 0.5 |
|  | PCaeC421 | 359 | 0.25 (0.19, 0.29) | 0.24 (0.20, 0.31) | 0.3 |
|  | PCaeC422 | 359 | 0.31 (0.24, 0.39) | 0.33 (0.28, 0.42) | 0.011 |
|  | PCaeC423 | 359 | 0.51 (0.39, 0.63) | 0.54 (0.46, 0.68) | 0.006 |
|  | PCaeC424 | 359 | 0.48 (0.36, 0.58) | 0.48 (0.40, 0.65) | 0.052 |
|  | PCaeC425 | 359 | 1.13 (0.91, 1.41) | 1.14 (0.96, 1.36) | 0.6 |
|  | PCaeC443 | 359 | 0.074 (0.058, 0.094) | 0.077 (0.062, 0.100) | 0.052 |
|  | PCaeC445 | 359 | 0.75 (0.61, 1.02) | 0.77 (0.63, 0.94) | > 0.9 |
|  | PCaeC446 | 359 | 0.82 (0.65, 1.02) | 0.81 (0.67, 1.02) | > 0.9 |
| Sphingomyelins | SMOHC141 | 359 | 3.54 (3.05, 4.40) | 4.11 (3.24, 4.96) | 0.006 |
|  | SMOHC161 | 359 | 2.82 (2.37, 3.38) | 3.06 (2.33, 3.63) | 0.2 |
|  | SMOHC221 | 359 | 18 (12, 27) | 21 (12, 31) | 0.1 |
|  | SMOHC222 | 359 | 17 (10, 27) | 17 (9, 29) | 0.6 |
|  | SMC160 | 359 | 120 (100, 140) | 125 (95, 146) | 0.6 |
|  | SMC161 | 359 | 17.0 (13.8, 20.5) | 17.9 (13.7, 20.6) | 0.6 |
|  | SMC180 | 359 | 25 (18, 30) | 25 (18, 31) | 0.8 |
|  | SMC181 | 359 | 12.9 (9.9, 15.6) | 12.2 (9.1, 14.9) | 0.15 |
|  | SMC202 | 359 | 0.45 (0.35, 0.54) | 0.46 (0.34, 0.55) | 0.7 |
|  | SMC240 | 359 | 27 (21, 34) | 31 (21, 38) | 0.081 |
|  | SMC241 | 359 | 99 (57, 143) | 96 (53, 145) | 0.5 |
| Others | C5OHC3DCM | 305 | 0.033 (0.027, 0.041) | 0.033 (0.025, 0.040) | 0.3 |
|  | ADMA | 359 | 0.40 (0.30, 0.50) | 0.34 (0.30, 0.40) | 0.005 |
|  | Creatinine_MS | 359 | 88 (70, 106) | 81 (65, 95) | 0.01 |
|  | PCaeC444 | 359 | 0.20 (0.16, 0.25) | 0.20 (0.17, 0.25) | 0.5 |
|  | SMOHC241 | 359 | 1.29 (1.09, 1.49) | 1.37 (1.06, 1.70) | 0.031 |
|  | SMC260 | 359 | 0.22 (0.18, 0.28) | 0.25 (0.17, 0.31) | 0.12 |
|  | SMC261 | 359 | 0.51 (0.41, 0.62) | 0.50 (0.38, 0.66) | 0.8 |
| *^1^* Numerical data are presented as medians (interquartile range), and categorical data are presented in terms of N (%). | | | | | |
| *^2^* The Wilcoxon rank-sum test is used for analyzing continuous variables; Pearson's chi-squared test is used for examining categorical variables for which expected cell counts are ≥ 5; and Fisher’s exact test is used for investigating categorical variables for which expected cell count is < 5. | | | | | |

**Supplementary table 2**. Features selected through recursive feature elimination with cross-validation for the six adopted models. SVM: support vector machine, XGBoost: extreme gradient boosting. Clinical factors were marked as gray background.

| **Logistic_Regression** | **SVM** | **DecisionTree** | **RandomForest** | **XGBoost** | **GradientBoost** |
| --- | --- | --- | --- | --- | --- |
| Body weight | C7DC | Trp | PCaaC366 | PCaaC366 | PCaaC366 |
| Diabetes mellitus | lysoPCaC204 | PCaeC361 | Uric Acid | Uric Acid | Uric Acid |
| Pro | Pro | High sensitive C-reactive protein | C7DC | C7DC | C7DC |
| High-density lipoprotein cholesterol | Asn | Val | lysoPCaC204 | Heart rate | lysoPCaC204 |
| Asn | PCaaC364 | C182 | Pro | lysoPCaC204 | PCaeC422 |
| Cit | C2 | Smoking | Cit | Pro | Pro |
| Lys | PCaaC362 | Arg | C161 | Asn | Asn |
| PCaaC364 | PCaeC322 | C9 | Kynurenine | Cit | Cit |
| Anti-hypertensive | SMC161 | Anti-hypertensive | PCaaC364 | C161 | C5DCC6OH |
| C2 | PCaeC302 | C2 | PCaeC322 | t4OHPro | C161 |
| PCaeC424 | C102 | Body mass index | SMC181 | PCaaC364 | t4OHPro |
| C4 | PCaeC401 | Kynurenine | SMC161 | Kynurenine | PCaeC425 |
| SMC181 | PCaeC364 | C4 | PCaeC302 | C2 | PCaaC364 |
| Ser | C6C41DC |  | lysoPCaC182 | PCaeC322 | Kynurenine |
| SMC180 | PCaaC403 |  | Creatinine | SMC181 | ADMA |
| Anti-lipid | PCaeC383 |  | PCaeC321 | Gln | C2 |
| PCaeC302 | Creatinine |  | PCaeC380 | C102 | SMC181 |
| Low-density lipoprotein cholesterol | Alcohol |  | Anti-diabetic | C6C41DC | Gln |
| C141 | PCaeC380 |  | C0 | PCaaC403 | C102 |
| C6C41DC | Anti-diabetic |  | C12 | His | C3DCC4OH |
| SMC241 | C0 |  | PCaaC323 | Creatinine | PCaeC402 |
| PCaeC383 | PCaeC444 |  | Hip size | PCaaC341 | lysoPCaC182 |
| C5 | C41 |  | SMOHC161 | C18 | PCaeC383 |
| Creatinine | SMOHC161 |  | PCaeC362 | Alcohol | Creatinine |
| Thr | C3 |  | C9 | PCaeC380 | PCaaC341 |
| Alcohol | C9 |  | C3 | C0 | C18 |
| Anti-diabetic | PCaaC422 |  | PCaaC422 | C12 | PCaeC321 |
| Trp | PCaeC382 |  | PCaeC420 | C16OH | PCaeC380 |
| C0 | lysoPCaC281 |  | PCaaC402 | PCaaC323 | Anti-diabetic |
| C41 | PCaaC360 |  | Val | Hip size | C0 |
| PCaaC406 | C161OH |  | lysoPCaC161 | SMOHC161 | C12 |
| PCaeC360 | Diastolic blood pressure |  | Diastolic blood pressure | C3 | PCaaC323 |
| SMOHC161 | Hypertension |  | SMC260 | PCaaC422 | Hip size |
| C3 | C142OH |  | C8 | PCaaC342 | SMOHC161 |
| PCaeC362 | PCaeC405 |  | Age | PCaeC420 | PCaeC362 |
| Sarcosine | C8 |  | PCaeC363 | PCaeC382 | C3 |
| PCaaC422 | C181OH |  | Body mass index | lysoPCaC281 | PCaaC342 |
| C10 | PCaaC365 |  | lysoPCaC260 | PCaaC360 | PCaeC420 |
| PCaaC300 | PCaaC424 |  | Diabetes mellitus | Val | PCaeC382 |
| Ala | Age |  | Glucose | lysoPCaC161 | PCaaC402 |
| lysoPCaC281 | Body mass index |  | Glu | Diastolic blood pressure | Val |
| PCaeC342 | SMOHC222 |  | Body weight | C181 | lysoPCaC161 |
| PCaaC360 | Diabetes mellitus |  | SMC202 | Hypertension | Diastolic blood pressure |
| PCaaC404 | C12DC |  | High-density lipoprotein cholesterol | SMC260 | C161OH |
| Diastolic blood pressure | Body weight |  | SDMA | C8 | C3OH |
| Hypertension | High-density lipoprotein cholesterol |  | Lys | PCaaC365 | Hypertension |
| C182 | Lys |  | PCaaC426 | Age | SMC260 |
| SMOHC221 | PCaeC424 |  | Anti-hypertensive | PCaeC363 | C162OH |
| Smoking | Anti-hypertensive |  | C121 | Body mass index | C8 |
| C142OH | C4 |  | PCaeC300 | lysoPCaC260 | Age |
| C8 | Ser |  | C4 | PCaeC421 | Body mass index |
| PCaeC405 | PCaaC343 |  | PCaaC281 | Diabetes mellitus | lysoPCaC260 |
| PCaaC424 | Anti-lipid |  | Ile | Glucose | PCaeC421 |
| PCaaC365 | SMC180 |  | Ser | Glu | SMOHC222 |
| Age | Low-density lipoprotein cholesterol |  | Anti-lipid | Body weight | Diabetes mellitus |
| Phe | PCaaC344 |  | PCaaC343 | SMC202 | C12DC |
| Body mass index | C141 |  | Low-density lipoprotein cholesterol | High-density lipoprotein cholesterol | Glucose |
| Kynurenine | SMC261 |  | Homocysteine | SDMA | C61 |
| lysoPCaC260 | PCaaC361 |  | PCaaC344 | Anti-hypertensive | Glu |
| SMOHC222 | C5 |  | C141 | C121 | PCaaC380 |
| C12DC | Thr |  | SMC261 | PCaeC300 | Body weight |
| Total cholesterol | Trp |  | PCaaC383 | C4 | SMC202 |
|  | lysoPCaC240 |  | SMC241 | PCaaC281 | alphaAAA |
|  | C142 |  | PCaaC361 | Ile | High-density lipoprotein cholesterol |
|  | PCaeC341 |  | C5 | Ser | SDMA |
|  | PCaaC363 |  | Tyr | Anti-lipid | Lys |
|  | PCaaC406 |  | PCaaC421 | SMC180 | Anti-hypertensive |
|  | PCaeC360 |  | Mean blood pressure | SMC240 | C121 |
|  | Sarcosine |  | SMOHC141 | Low-density lipoprotein cholesterol | PCaeC300 |
|  | Triglyceride |  | Trp | Gly | C4 |
|  | Orn |  | High sensitive C-reactive protein | PCaeC423 | Ile |
|  | C10 |  | PCaaC240 | Homocysteine | Ser |
|  | PCaaC300 |  | PCaeC343 | C141 | Anti-lipid |
|  | Ala |  | PCaeC446 | SMC261 | SMC180 |
|  | PCaaC404 |  | PCaeC386 | PCaaC383 | SMC240 |
|  | lysoPCaC170 |  | PCaaC406 | Arg | Low-density lipoprotein cholesterol |
|  | Smoking |  | SMOHC241 | C51DC | Gly |
|  | C14 |  | Met | SMC241 | PCaeC423 |
|  | C182 |  | Triglyceride | Mean blood pressure | C141 |
|  | Phe |  | Sarcosine | SMOHC141 | SMC261 |
|  | Kynurenine |  | PCaeC340 | C51 | PCaaC383 |
|  | SMOHC221 |  | C10 | lysoPCaC261 | Arg |
|  | Total cholesterol |  | PCaaC425 | Trp | C51DC |
|  |  |  | Creatinine_MS | lysoPCaC240 | SMC241 |
|  |  |  | PCaeC342 | PCaeC361 | PCaaC361 |
|  |  |  | C101 | High sensitive C-reactive protein | C5 |
|  |  |  | PCaeC445 | PCaeC343 | C51 |
|  |  |  | C182 | PCaeC446 | Trp |
|  |  |  | Systolic blood pressure | PCaeC386 | lysoPCaC240 |
|  |  |  | Leu | PCaaC406 | PCaeC361 |
|  |  |  | Asp | SMOHC241 | High sensitive C-reactive protein |
|  |  |  | Phe | Met | PCaaC240 |
|  |  |  | lysoPCaC203 | Triglyceride | PCaeC343 |
|  |  |  | PCaaC384 | PCaeC360 | PCaeC446 |
|  |  |  | Total cholesterol | Sarcosine | PCaaC363 |
|  |  |  |  | PCaeC340 | PCaeC386 |
|  |  |  |  | Orn | PCaaC406 |
|  |  |  |  | C10 | SMOHC241 |
|  |  |  |  | PCaaC385 | Met |
|  |  |  |  | PCaaC300 | Triglyceride |
|  |  |  |  | Creatinine_MS | PCaeC360 |
|  |  |  |  | Ala | Sarcosine |
|  |  |  |  | PCaeC342 | PCaeC340 |
|  |  |  |  | C16 | Orn |
|  |  |  |  | C101 | C10 |
|  |  |  |  | PCaaC404 | PCaaC385 |
|  |  |  |  | PCaeC385 | PCaaC300 |
|  |  |  |  | Smoking | Creatinine_MS |
|  |  |  |  | C182 | Ala |
|  |  |  |  | Systolic blood pressure | PCaeC342 |
|  |  |  |  | Asp | C101 |
|  |  |  |  | Phe | PCaaC404 |
|  |  |  |  | lysoPCaC203 | Body height |
|  |  |  |  | PCaaC384 | Smoking |
|  |  |  |  | Total cholesterol | C182 |
|  |  |  |  |  | PCaeC385 |
|  |  |  |  |  | Systolic blood pressure |
|  |  |  |  |  | Leu |
|  |  |  |  |  | Asp |
|  |  |  |  |  | Phe |
|  |  |  |  |  | lysoPCaC203 |
|  |  |  |  |  | PCaaC384 |
|  |  |  |  |  | Total cholesterol |

**Supplementary table 3**. The 62 features selected through recursive feature elimination with cross-validation for the Logistic regression model

| **Clinical factors** | **Metabolites** |
| --- | --- |
| Age | Ala |
| Alcohol | Asn |
| Body mass index | C0 |
| Body weight | C10 |
| Total cholesterol | C12DC |
| Creatinine | C141 |
| Diastolic blood pressure | C142OH |
| Diabetes mellitus | C182 |
| Anti-diabetic | C2 |
| High-density lipoprotein cholesterol | C3 |
| Hypertension | C4 |
| Anti-hypertensive | C41 |
| Low-density lipoprotein cholesterol | C5 |
| Anti-lipid | C6C41DC |
| Smoking | C8 |
|  | Cit |
|  | Kynurenine |
|  | Lys |
|  | lysoPCaC260 |
|  | lysoPCaC281 |
|  | PCaaC300 |
|  | PCaaC360 |
|  | PCaaC364 |
|  | PCaaC365 |
|  | PCaaC404 |
|  | PCaaC406 |
|  | PCaaC422 |
|  | PCaaC424 |
|  | PCaeC302 |
|  | PCaeC342 |
|  | PCaeC360 |
|  | PCaeC362 |
|  | PCaeC383 |
|  | PCaeC405 |
|  | PCaeC424 |
|  | Phe |
|  | Pro |
|  | Sarcosine |
|  | Ser |
|  | SMC180 |
|  | SMC181 |
|  | SMC241 |
|  | SMOHC161 |
|  | SMOHC221 |
|  | SMOHC222 |
|  | Thr |
|  | Trp |

.

**Supplementary table 4**. The 27 shared features among LR, SVM, RF, Xgboost, and Gradient boosting models. LR: logistic regression, RF: random forest, SVM: support vector machine, XGBoost: extreme gradient boosting.

| **Clinical factors** | **Metabolites** |
| --- | --- |
| Age | C0 |
| Body mass index | C10 |
| Body weight | C141 |
| Total cholesterol | C182 |
| Creatinine | C3 |
| Diabetes mellitus | C4 |
| Diastolic blood pressure | C8 |
| High-density lipoprotein cholesterol | Kynurenine |
| Anti-hypertensive | PCaaC364 |
| Anti-lipid | PCaaC406 |
| Low-density lipoprotein cholesterol | Phe |
|  | Pro |
|  | Sarcosine |
|  | Ser |
|  | SMOHC161 |
|  | Trp |

**Supplementary table 5.** Pathway analysis on the 47 metabolites by using Metaboanalyst v5.0

| **Pathway Name** | **Match Status** | ***p*-value** | **-log(*p*)** | **Holm *p*** | **FDR** | **Impact** | **Details** |
| --- | --- | --- | --- | --- | --- | --- | --- |
| Aminoacyl-tRNA biosynthesis | 8/48 | **6.62E-07** | 6.1791 | 5.56E-05 | **5.56E-05** | 0.1667 | hsa00970 |
| Glycine, serine and threonine metabolism | 3/33 | **1.82E-02** | 1.7408 | 1.00E+00 | 6.43E-01 | 0.3100 | hsa00260 |
| Glycerophospholipid metabolism | 3/36 | **2.30E-02** | 1.6391 | 1.00E+00 | 6.43E-01 | 0.1205 | hsa00564 |
| Ether lipid metabolism | 2/20 | **4.58E-02** | 1.3393 | 1.00E+00 | 7.86E-01 | 0.2651 | hsa00565 |
| Sphingolipid metabolism | 2/21 | 5.01E-02 | 1.3004 | 1.00E+00 | 7.86E-01 | 0.0000 | hsa00600 |
| Phenylalanine, tyrosine and tryptophan biosynthesis | 1/4 | 6.79E-02 | 1.1679 | 1.00E+00 | 7.86E-01 | 0.5000 | hsa00400 |
| Lysine degradation | 2/25 | 6.86E-02 | 1.164 | 1.00E+00 | 7.86E-01 | 0.0000 | hsa00310 |
| Alanine, aspartate and glutamate metabolism | 2/28 | 8.37E-02 | 1.0774 | 1.00E+00 | 7.86E-01 | 0.0000 | hsa00250 |
| Linoleic acid metabolism | 1/5 | 8.42E-02 | 1.0746 | 1.00E+00 | 7.86E-01 | 0.0000 | hsa00591 |
| Valine, leucine and isoleucine biosynthesis | 1/8 | 1.31E-01 | 0.88131 | 1.00E+00 | 1.00E+00 | 0.0000 | hsa00290 |
| Tryptophan metabolism | 2/41 | 1.58E-01 | 0.80026 | 1.00E+00 | 1.00E+00 | 0.2372 | hsa00380 |
| Biotin metabolism | 1/10 | 1.62E-01 | 0.79159 | 1.00E+00 | 1.00E+00 | 0.0000 | hsa00780 |
| Phenylalanine metabolism | 1/10 | 1.62E-01 | 0.79159 | 1.00E+00 | 1.00E+00 | 0.3571 | hsa00360 |
| alpha-Linolenic acid metabolism | 1/13 | 2.05E-01 | 0.68836 | 1.00E+00 | 1.00E+00 | 0.0000 | hsa00592 |
| Arginine biosynthesis | 1/14 | 2.19E-01 | 0.65973 | 1.00E+00 | 1.00E+00 | 0.2284 | hsa00220 |
| Selenocompound metabolism | 1/20 | 2.98E-01 | 0.52596 | 1.00E+00 | 1.00E+00 | 0.0000 | hsa00450 |
| Glyoxylate and dicarboxylate metabolism | 1/32 | 4.33E-01 | 0.36312 | 1.00E+00 | 1.00E+00 | 0.0423 | hsa00630 |
| Cysteine and methionine metabolism | 1/33 | 4.43E-01 | 0.35314 | 1.00E+00 | 1.00E+00 | 0.0218 | hsa00270 |
| Arachidonic acid metabolism | 1/36 | 4.73E-01 | 0.32544 | 1.00E+00 | 1.00E+00 | 0.0000 | hsa00590 |
| Arginine and proline metabolism | 1/38 | 4.91E-01 | 0.30863 | 1.00E+00 | 1.00E+00 | 0.0778 | hsa00330 |

# References

1. PENG, C.-Y.J., K.L. LEE, and G.M. INGERSOLL, *An Introduction to Logistic Regression Analysis and Reporting.* The Journal of Educational Research, 2002. **96**: p. 3-14.

2. Vapnik, C.a., *Support vector networks.* Machine Learning, 1995. **20**(3): p. 273–297.

3. Chang, W., et al., *A Machine-Learning-Based Prediction Method for Hypertension Outcomes Based on Medical Data. LID - 10.3390/diagnostics9040178 [doi] LID - 178.* 2019(2075-4418 (Print)).

4. Badr HSSINA, A.M., Hanane EZZIKOURI and Mohammed ERRITALI, *A comparative study of decision tree ID3 and C4.5.* International Journal of Advanced Computer Science and Applications(IJACSA), 2014. **Special Issue on Advances in Vehicular Ad Hoc Networking and Applications 2014**.

5. Bauer, E. and R. Kohavi, *An Empirical Comparison of Voting Classification Algorithms: Bagging, Boosting, and Variants.* Machine Learning, 1999. **36**(1): p. 105-139.

6. Díaz-Uriarte, R. and S. Alvarez de Andrés, *Gene selection and classification of microarray data using random forest.* 2006(1471-2105 (Electronic)).

7. Pal, M., *Random forest classifier for remote sensing classification.* International Journal of Remote Sensing, 2005. **26**(1): p. 217-222.

8. Gray, K.R., et al., *Random forest-based similarity measures for multi-modal classification of Alzheimer's disease.* 2013(1095-9572 (Electronic)).

9. Chen, T. and C. Guestrin *XGBoost: A Scalable Tree Boosting System*. 2016. arXiv:1603.02754.

10. Nielsen, D. *Tree Boosting With XGBoost - Why Does XGBoost Win "Every" Machine Learning Competition?* 2016.

11. Le, N.Q., T.T. Nguyen, and Y.Y. Ou, *Identifying the molecular functions of electron transport proteins using radial basis function networks and biochemical properties.* 2017(1873-4243 (Electronic)).

12. Berrar, D., *Cross-Validation*. 2018.

13. Govindarajan, M. and R. Chandrasekaran, *Evaluation of k-nearest neighbor classifier performance for direct marketing.* Expert Systems with Applications, 2010. **37**(1): p. 253-258.
